# Honokiol potentiates low-dose sulfamonomethoxine sodium against antimicrobial-resistant AHPND-causing *Vibrio parahaemolyticus* through bacterial membrane disruption and host recovery

**DOI:** 10.3389/fimmu.2026.1882728

**Published:** 2026-06-30

**Authors:** Yihuan Chen, Liyuan Yi, Le Zhang, Yiming Zheng, Chen Chen, Yaohua Wang, Shilin Shang, Maocang Yan, Qianjin Zhou, Jiong Chen

**Affiliations:** 1 Laboratory of Biochemistry and Molecular Biology, School of Marine Sciences, Ningbo University, Ningbo, China; 2 Key Laboratory of Aquacultural Biotechnology of Ministry of Education, Ningbo University, Ningbo, China; 3 Zhejiang Mariculture Research Institute, Zhejiang Key Laboratory of Coastal Biological Germplasm Resources Conservation and Utilization, Wenzhou, China

**Keywords:** acute hepatopancreatic necrosis disease, antibiotic potentiation, antimicrobial resistance, gut microbiota, honokiol, host immune recovery, sulfamonomethoxine sodium, *Vibrio parahaemolyticus*

## Abstract

Acute hepatopancreatic necrosis disease (AHPND), caused by *Vibrio parahaemolyticus*, poses a severe threat to global shrimp aquaculture. Reduced susceptibility of aquaculture-associated bacterial pathogens to conventional antibiotics, together with concerns regarding antimicrobial input and environmental exposure, highlights the need for dose-sparing therapeutic strategies. This study investigated the antibiotic-potentiating activity of honokiol (HKL), a plant-derived lignan, in combination with sulfamonomethoxine sodium (SMM-Na) against AHPND. Checkerboard and time-kill assays demonstrated that sub-inhibitory HKL concentrations (32–64 μg/mL) markedly enhanced SMM-Na activity, reducing its minimum inhibitory concentration by 8–32-fold and achieving bactericidal effects in combination. The SMM-Na + HKL combination also significantly inhibited biofilm formation and compromised bacterial membrane integrity, as evidenced by enhanced propidium iodide uptake and scanning electron microscopy, while inducing severe ATP depletion. In *Penaeus vannamei* challenged with *V. parahaemolyticus*, dietary administration of low-dose SMM-Na (16 mg/kg) and HKL (32 mg/kg) yielded the highest survival rate (86.6%), substantially reduced hepatopancreatic bacterial load, attenuated histopathological damage, and restored total hemocyte counts. The treatment also upregulated a broad spectrum of immune-related genes (e.g., *Alf*, *Tlr*, *Lec*, *Crustin*, *Lzm*, *CatB*) in the hepatopancreas and mucosal barrier genes (e.g., *Muc-1*, *Muc-4*, *Muc-5AC*, *Muc-19*) in the intestine. Furthermore, 16S rRNA sequencing indicated that the combination therapy was associated with treatment-related shifts in the intestine microbiota, including increased alpha diversity indices, reduced *Vibrio* abundance, and altered predicted KEGG functional profiles. Collectively, our results identify HKL as a natural-origin antibiotic potentiator that enhances the efficacy of low-dose SMM-Na against AHPND-causing *V. parahaemolyticus*. These findings provide proof-of-concept evidence for a dose-sparing combination strategy that may reduce therapeutic SMM-Na input while supporting pathogen control and host recovery in shrimp aquaculture.

## 1 Introduction

Aquaculture is a crucial pillar of global animal protein supply, with the Pacific white shrimp (*Penaeus vannamei*) being one of the most important economic shrimp species (1). However, the intensive farming model has led to frequent outbreaks of bacterial diseases, among which vibriosis caused by bacteria of the genus *Vibrio* is a major biological threat constraining the healthy development of the industry (2).

These pathogens are widely distributed and can infect various hosts such as fish, shrimp, and shellfish. They often secrete a variety of toxins (e.g., hemolysins, proteases, MARTX toxins) to disrupt host tissues and immune defenses, resulting in high mortality rates (3). Particularly noteworthy are *Vibrio* spp. carrying the *pirA/B* toxin-associated virulence plasmid (e.g., *V. parahaemolyticus*), which can cause acute hepatopancreatic necrosis disease (AHPND). This disease is characterized by sudden onset and high lethality, and since its outbreak in 2009, it has caused significant economic losses to the global shrimp farming industry (4, 5). Currently, the control of such bacterial diseases still heavily relies on antibiotics. However, long-term and frequent use has led to increasingly severe antimicrobial resistance (AMR) in *Vibrio* spp., even the emergence of multidrug-resistant strains (6). This not only weakens therapeutic efficacy but also poses environmental and food safety risks (3). Therefore, exploring novel strategies to improve the efficacy of existing antibiotics while reducing the required therapeutic dose and environmental antimicrobial input is crucial for the sustainable development of aquaculture.

To improve antibiotic efficacy while reducing therapeutic antibiotic input, the development of antibiotic adjuvants has emerged as a promising direction. This strategy does not abandon antibiotics entirely but aims to enhance the antibacterial performance of existing antibiotics by combining them with other compounds. In this context, the main practical value of antibiotic adjuvants is not necessarily to prove direct suppression of resistance emergence, but to achieve effective pathogen control with lower antibiotic input and improved therapeutic performance. Plant-derived bioactive substances are considered promising adjuvant candidates due to their wide availability, multi-target effects, and good environmental compatibility (7). Studies have shown that combinations of various plant extracts or monomers with antibiotics can produce enhanced antibacterial effects. For example, Naringenin reverses colistin resistance in colistin-resistant Gram-negative bacteria *in vitro* and *in vivo* (8); curcumin significantly reduces the minimum inhibitory concentration (MIC) of methicillin-resistant *Staphylococcus aureus* (MRSA) to various antibiotics (e.g., oxacillin, ampicillin), thereby improving antibacterial susceptibility under experimental conditions (9). These successful cases provide a theoretical basis and practical reference for using plant-derived compounds as antibiotic adjuvants to control aquatic pathogens.

Sulfamonomethoxine sodium (SMM-Na) is a long-acting sulfonamide antibacterial agent approved for use in aquaculture and holds certain application value in preventing and treating *Vibrio* infections (10). However, with prolonged use, resistance to SMM-Na has also emerged, limiting its efficacy (10). Currently, systematic research on utilizing plant-derived bioactive substances as adjuvants to enhance SMM-Na activity and support dose-sparing therapeutic strategies is still relatively scarce. Honokiol (HKL), a natural biphenolic compound extracted from the traditional Chinese medicine *Magnolia officinalis*, has been reported to possess various biological activities such as antimicrobial, anti-inflammatory, and antioxidant properties (11–19). Preliminary studies indicate that HKL has inhibitory effects against various pathogens and can disrupt biofilms (12). However, whether HKL can serve as an effective antibiotic adjuvant, particularly in combination with SMM-Na to enhance antibacterial activity against the important aquaculture pathogen *V. parahaemolyticus*, and the underlying mechanisms remain unclear.

Accordingly, this study systematically investigated the potential of HKL as an antibiotic adjuvant to SMM-Na against *V. parahaemolyticus* and in controlling AHPND. By integrating *in vitro* and *in vivo* experiments, this study first evaluated the *in vitro* synergistic antibacterial activity of HKL and SMM-Na, including their effects on bacterial susceptibility, biofilm formation, cell membrane integrity, and energy metabolism. Subsequently, the *in vivo* protective efficacy of this combination was validated in a *P. vannamei* challenge model, with a focus on its improvement of survival rate, hepatopancreatic bacterial load, immune recovery, and histopathological damage. Furthermore, considering the close link between intestinal microbiota homeostasis and host health, this study further analyzed the impact of the combined treatment on the structure of the intestinal microbial community and its predicted metabolic functions. This study aims to comprehensively evaluate the potential of HKL as a functional adjuvant to enhance the efficacy of SMM-Na and to provide proof-of-concept evidence for a dose-sparing combination strategy against AHPND-causing *V. parahaemolyticus*, providing new insights for developing integrated control strategies that improve antibacterial efficacy while reducing therapeutic antibiotic input in aquaculture.

## 2 Materials and methods

### 2.1 Antimicrobial susceptibility test

*V. parahaemolyticus* DX190406, kindly provided by Zhejiang Mariculture Research Institute, was used throughout this study. The strain was isolated from *P. vannamei* exhibiting typical symptoms of AHPND at a shrimp farm in Ninghai, Zhejiang Province, China. Its whole-genome sequence has been determined and deposited in the NCBI GenBank database under accession number CP187480–CP187485. This isolate was selected as a clinically relevant AHPND-associated strain for evaluating the antibiotic-potentiating activity of HKL in combination with SMM-Na.

Antimicrobial susceptibility was evaluated by determining the minimum inhibitory concentrations (MICs) of a panel of antibiotics commonly used in aquaculture, together with the phytogenic compound honokiol (HKL). The tested antibiotics included florfenicol, enrofloxacin, doxycycline hydrochloride, and sulfamonomethoxine sodium (SMM-Na) (Shanghai Macklin Biochemical Technology Co., Ltd., China). Stock solutions of all antibiotics (5120 μg/mL) were prepared according to the manufacturers’ instructions. HKL (purity ≥ 98%) was purchased from Shanghai Aladdin Biochemical Technology Co., Ltd. (China), dissolved in dimethyl sulfoxide (DMSO) and stored at −20 °C until use.

MICs were determined using the broth microdilution method in 2216E liquid medium. Briefly, two-fold serial dilutions of each antibiotic or HKL were prepared in sterile 96-well microtiter plates. Overnight cultures of *V. parahaemolyticus* DX190406 grown in 2216E broth at 28 °C were adjusted to a final concentration of approximately 1.5 × 10^6^ CFU/mL and added to each well. For HKL-containing treatments, solvent controls containing the corresponding concentration of DMSO were included to exclude solvent-associated effects. Plates were incubated at 28 °C for 18 h without shaking. The MIC was defined as the lowest concentration of a compound that completely inhibited visible bacterial growth. All assays were performed independently in triplicate. MIC values were interpreted with reference to the latest guidelines of the Clinical and Laboratory Standards Institute (CLSI), where applicable. In cases where CLSI breakpoints were not available for *Vibrio* spp. or specific antimicrobial agents, MIC results were reported descriptively.

### 2.2 Checkerboard assays

The synergistic activity between honokiol (HKL) and SMM-Na was evaluated using a checkerboard assay, performed with slight modifications according to a previously described method (20). Briefly, the antibiotic SMM-Na was subjected to twofold serial dilutions along the vertical (Y) axis of a 96-well microplate, while HKL was diluted similarly along the horizontal (X) axis. Subsequently, bacterial inoculum was added to achieve a final inoculum density of approximately 1.5 × 10^6^ colony-forming units per milliliter (CFU/mL). Wells containing bacteria without antimicrobial treatment and wells containing medium only were included as growth and sterility controls, respectively. Following incubation at 28 °C for 18 h, bacterial growth was assessed. The fractional inhibitory concentration index (FICI) was calculated to determine the interaction between the two agents using the formula (21): FICI = (MIC of SMM-Na in combination/MIC of SMM-Na alone) + (MIC of HKL in combination/MIC of HKL alone). Synergy was defined as FICI of ≤ 0.5; additivity as 0.5 < FICI ≤ 1; indifference as 1 < FICI ≤ 4; and antagonism as an FICI > 4 (21). All tests were performed in triplicate.

### 2.3 Growth curve determination

A single colony of *V. parahaemolyticus* DX190406 was inoculated into 2216E liquid medium and cultured with shaking at 180 rpm and 28 °C for 12 h. The bacterial culture was then diluted to an initial density of approximately 1.5 × 10^6^ CFU/mL, and treated with SMM-Na, HKL, or their combination at the specified concentrations. Untreated bacteria were included as a control. Growth kinetics were monitored in a 96-well microplate incubated at 28 °C by recording the optical density at 600 nm (OD_600_) at 1 h intervals using a microplate reader (22).

### 2.4 Time-kill assays

Time-kill assays were conducted based on a published protocol with modifications (23). Briefly, bacterial suspensions adjusted to approximately 1.5 × 10^6^ CFU/mL were treated with SMM-Na, HKL, or the SMM-Na + HKL combination. Cultures containing bacterial cells without drug treatment served as the growth control. At predetermined time points (0, 10, and 30 min, as well as 3, 6, 9, 12, and 24 h), 200 μL of bacterial suspension was collected from each treatment and washed once with a ten-fold volume of sterile PBS to minimize residual drug carry-over. The cells were pelleted by centrifugation and resuspended in 200 μL of antibiotic-free 2216E broth. The recovered suspensions were subjected to ten-fold serial dilution when necessary, and 50 μL aliquots were spread onto antibiotic-free 2216E agar plates. Appropriate serial dilutions were prepared to obtain countable colony numbers, approximately 30–300 colonies per plate, whenever possible. For samples in which no colonies were detected after plating 50 μL of the undiluted recovered suspension, the remaining recovered suspension was subsequently plated in full to further confirm the absence of culturable bacteria. Plates were incubated overnight at 28 °C, and colonies were enumerated and expressed as CFU/mL. A reduction of ≥3 log_10_ in the colony count at 24 h by the drug combination, compared to the most active single agent, was defined as indicative of bactericidal activity (24). When no colonies were detected after plating both the initial aliquot and the remaining recovered suspension, the result was recorded as below the detection limit of the assay. The assay was independently repeated three times.

### 2.5 Inhibition of biofilm formation assay

The combined effect of HKL and SMM-Na on biofilm formation was investigated using a standard crystal violet (CV) staining assay, adapted from a previous study with modifications (25). Overnight cultures of *V. parahaemolyticus* were adjusted to a density of approximately 1.5 × 10^6^ CFU/mL in fresh 2216E medium. Aliquots (100 μL) of the adjusted bacterial suspension were dispensed into individual wells of a sterile 96-well polystyrene microplate. To these wells, SMM-Na, HKL, or their combination were added at predetermined concentrations. Wells containing only the bacterial suspension served as the untreated control. The plates were incubated at 28 °C for 24 h to allow biofilm formation. Subsequently, the suspended cells and medium were discarded from the wells, and the adherent biofilms were then gently washed twice with 200 μL sterile distilled water to remove non-adherent cells and allowed to air dry completely. The biofilms were stained with 150 μL of a 0.4% (w/v) CV solution per well for 20 min at room temperature. After incubation, the unbound CV was washed away with sterile distilled water, and the wells were air-dried. After drying, the CV bound to the biofilm was solubilized by adding 200 μL of 33% (v/v) glacial acetic acid to each well with gentle shaking for 10 min. The absorbance of the solubilized dye was then measured at 570 nm using a microplate reader to determine the relative biofilm biomass. All tests were performed in triplicate.

### 2.6 Eradication of pre-formed biofilms after different pre-formation periods

The efficacy of the agents against established biofilms was evaluated following a published protocol with modifications (26). *V. parahaemolyticus* suspensions were adjusted to approximately 1.5 × 10^6^ CFU/mL in 2216E medium. Aliquots (200 μL) were added to the wells of a 96-well microtiter plate and incubated at 28 °C for 6, 9, 12, or 24 h to allow for biofilm formation. After incubation, the suspended cells and spent medium were carefully aspirated. The adhered, pre-formed biofilms were then gently washed twice with 200 μL of sterile PBS. Fresh medium (200 μL) containing HKL, SMM-Na, or their combination at the desired concentrations was added to the washed biofilm-containing wells. The plate was incubated for an additional 12 h at 28 °C. Following this treatment period, the biofilm biomass remaining in each well was quantified using the CV staining method as described in Section 2.5. The experiment was performed independently three times.

### 2.7 Assessment of cell membrane permeability

The bacterial membrane permeability was evaluated using dual fluorescent staining with Hoechst 33342 and propidium iodide (PI), which allows discrimination between cells with intact membranes and those with compromised membrane integrity (25).

Overnight cultures of *V. parahaemolyticus* were harvested and adjusted to a density of approximately 1.5 × 10^6^ CFU/mL in fresh 2216E medium. The bacterial suspensions were then treated with SMM-Na, HKL, or their combination at the specified concentrations and incubated at 28 °C for 10 min. Untreated bacteria served as the control. After treatment, bacterial cells were collected by gentle centrifugation, washed once with sterile PBS to remove residual compounds, and resuspended in PBS. Following treatment, the cells were collected by gentle centrifugation, washed once with PBS, and resuspended in PBS. The cell suspension was then incubated with Hoechst 33342 (final concentration, 10 μg/mL) for 20 min at room temperature in the dark. After incubation, cells were collected again by gentle centrifugation and resuspended in 200 µL of PBS, followed by the addition of propidium iodide (PI; final concentration, 10 μg/mL). After staining with PI for 20 min at room temperature in the dark, cells were washed with PBS to remove excess dye, resuspended in a small volume of PBS, immobilized on a glass slide, and examined using a fluorescence microscope. Hoechst 33342, a membrane-permeable dye, stained the nucleic acids of all bacterial cells and emitted blue fluorescence, whereas PI selectively penetrated cells with compromised membrane integrity and emitted red fluorescence. Fluorescence images were captured to qualitatively assess changes in membrane permeability following treatment with SMM-Na, HKL, or their combination, based on differences in fluorescence distribution and staining patterns (25). The assay was independently repeated three times.

### 2.8 Scanning electron microscopy

Bacterial suspensions of *V. parahaemolyticus* at approximately 1.5 × 10^6^ CFU/mL were treated with HKL (64 μg/mL), SMM-Na (32 μg/mL), or their combination for 30 min with shaking at 28 °C. After treatment, the cells were harvested by centrifugation (6000 rpm for 5 min at 4 °C) and washed twice with sterile PBS (0.01 M, pH 7.4) (27). The bacterial pellets were gently resuspended in a small volume of fresh PBS. The bacterial suspensions were then fixed with an equal volume of 2.5% glutaraldehyde (prepared in 0.1 M PBS, pH 7.4) at 4 °C for at least 4 h. After fixation, the samples were rinsed three times with PBS by centrifugation (6000 rpm for 5 min at 4 °C). Subsequently, the fixed cells were progressively dehydrated in a graded ethanol series (typically 30%, 50%, 70%, 80%, 90%, 95%, and 100% ethanol, v/v), with each step lasting 10–15 minutes. Following dehydration, the samples were placed on clean glass coverslips, air-dried, and sputter-coated with a thin layer of gold. Morphological alterations of the bacterial cells were examined using a scanning electron microscope (Hitachi S-3400, Tokyo, Japan) (27).

### 2.9 Intracellular ATP determination

Intracellular ATP levels in *V. parahaemolyticus* DX190406 were quantified using an enhanced ATP assay kit (Beyotime, Shanghai, China) according to the manufacturer’s instructions (7). Overnight bacterial cultures were diluted to approximately 1.5 × 10^6^ CFU/mL in fresh 2216E medium. Aliquots of the bacterial cultures were treated with SMM-Na, HKL, or their combination at 28 °C. At specified time points (0, 5, 10, 15, and 20 min), 100 μL of the treated bacterial cultures were immediately transferred to pre-chilled microcentrifuge tubes and centrifuged at 6000 rpm for 5 min at 4 °C. Bacterial pellets were rapidly lysed by adding 100 μL of lysis buffer (Beyotime, Shanghai, China), and all subsequent steps were performed on ice. To ensure complete contact between the lysis buffer and the cells, the mixture was thoroughly mixed by repeated pipetting until a homogeneous suspension was achieved. The lysate was then centrifuged at 12,400 rpm for 5 min at 4 °C. Thirty microliters of the supernatant were transferred to a white 96-well plate, and 100 μL of ATP detection working solution was added (final volume, 130 μL). Luminescence was measured immediately after mixing using a microplate reader (Tecan Infinite M200, Tecan Austria GmbH, Groedig, Austria). Each treatment was independently repeated three times.

### 2.10 Reactive oxygen species variation

Intracellular ROS levels were measured using the fluorescent probe 2’,7’-dichloro-dihydrofluorescein diacetate (DCFH-DA) (7). *V. parahaemolyticus* DX190406 cultures were adjusted to approximately 1.5 × 10^6^ CFU/mL. Bacterial suspensions were first incubated with DCFH-DA at a final concentration of 10 μmol/L at 28 °C for 20 min in the dark to allow probe loading. After incubation, the cells were washed twice with PBS by centrifugation at 6000 rpm for 5 min to remove excess probe and then resuspended in PBS. Subsequently, 190 μL of the probe-loaded bacterial suspension was transferred to each well of a 96-well plate and mixed with 10 μL of SMM-Na, HKL, or their combination at the indicated concentrations. Untreated bacteria served as the negative control, and ROSUP was included as a positive control. The fluorescence intensity was continuously measured for 20 min after treatment using a microplate reader at 2-min intervals, with the excitation wavelength set at 488 nm and the emission wavelength at 525 nm. The assay was independently repeated three times.

### 2.11 Experimental animals and feeding conditions

Healthy Pacific white shrimp (*P. vannamei*) with an average body length of 4.0 ± 0.33 cm and body weight of 1.05 ± 0.27 g were obtained from the mariculture farm of Zhejiang Mariculture Research Institute. Prior to experiments, shrimp were acclimatized for one week in twelve 300-L fiberglass tanks. They were fed a commercial diet twice daily (09:00 and 21:00) at a feeding rate of 0.5% of body weight. The rearing water was sand-filtered natural seawater, with 50% replaced daily. Water quality parameters were maintained as follows: temperature 28 ± 0.5 °C, pH 7.8–8.2, salinity approximately 21‰, and dissolved oxygen around 6 mg/L. All tanks were sterilized before use. Shrimp were confirmed to be free of *V. parahaemolyticus* by culture on thiosulfate-citrate-bile salts-sucrose (TCBS) agar and 16S rRNA gene PCR prior to the challenge experiments. Only apparently healthy shrimp with normal activity and feeding behavior were used for subsequent experiments.

### 2.12 Infection model establishment and dose-finding screening based on survival rate

#### 2.12.1 Establishment of *V. parahaemolyticus* challenge model

The median lethal dose (LD_50_ of *V. parahaemolyticus* strain DX190406) for *P. vannamei* was determined by a preliminary bioassay. Shrimp were immersed in bacterial suspensions at various concentrations for 15 min and then monitored for 120 h. The 120-h LD_50_ was calculated to be approximately 6.0 × 10^7^ CFU/mL using the Reed-Muench method (28). For the formal challenge, shrimp were first immersed in a high-density bacterial suspension (6.0 × 10^8^ CFU/mL) for 15 min to ensure initial exposure. They were then transferred to 30-L experimental tanks, where the waterborne bacterial concentration was maintained at the LD_50_ level (6.0 × 10^7^ CFU/mL) throughout the trial period. Shrimp in the negative control group were subjected to the same procedure using sterile Tryptic Soy Broth (TSB). All experimental procedures involving *P. vannamei* were conducted in accordance with accepted ethical principles for the care and use of aquatic animals in research. In accordance with institutional policy at Ningbo University, formal ethical review is not required for studies involving aquatic invertebrates such as *P. vannamei*, as these animals are not within the formal review scope of the institutional animal ethics committee no project-specific animal ethics approval number was issued for this study. Nevertheless, all experiments followed the 3Rs principles (Replacement, Reduction, and Refinement) and were conducted with reference to relevant national and local regulations (including Zhejiang provincial government order No. 263). Humane endpoints were applied to minimize suffering, and moribund shrimp were humanely euthanized by rapid ice-water immersion (ice slurry).

#### 2.12.2 Experimental design and drug administration for screening

To screen for the most effective drug combination ratio based on survival rate, a total of 630 shrimp were randomly distributed into seven groups (30 shrimp per tank, with three replicate tanks per group):

NC: Negative control (unchallenged), fed a commercial diet coated with peanut oil.
PC: Positive control (challenged), fed a commercial diet coated with peanut oil.
SMM-Na: Challenged, fed a diet containing 80 mg/kg SMM-Na.
HKL: Challenged, fed a diet containing 64 mg/kg HKL.
Combination A: Challenged, fed a diet containing 8 mg/kg SMM-Na and 64 mg/kg HKL.
Combination B: Challenged, fed a diet containing 16 mg/kg SMM-Na and 32 mg/kg HKL.
Combination C: Challenged, fed a diet containing 16 mg/kg SMM-Na and 64 mg/kg HKL.

Drugs were dissolved in peanut oil and uniformly coated onto the commercial feed pellets. The control diets were coated with the same amount of peanut oil. The medicated diets were administered immediately after challenge, twice daily for 10 days. Thirty percent of the rearing water was replaced with fresh seawater every three days. The tested dietary doses were selected based on the approved or commonly used aquaculture dose range of SMM-Na, the preliminary survival-based screening design, and the aim of evaluating whether HKL could support a lower SMM-Na input under challenge conditions.

#### 2.12.3 Survival analysis

Cumulative mortality in each group was recorded daily for 10 days. The survival rate was calculated as follows: Survival rate (%) = (initial numbers of shrimps − cumulative number of dead shrimps)/initial numbers of shrimps × 100%. Based on the results, Combination B (SMM-Na 16 mg/kg + HKL 32 mg/kg) yielded the highest survival rate and was selected as the representative low-dose combination treatment for the subsequent therapeutic efficacy experiment.

### 2.13 Therapeutic efficacy evaluation experiment

#### 2.13.1 Experimental design for efficacy assessment

This experiment was conducted to comprehensively evaluate the therapeutic efficacy and associated host responses of the low-dose combination identified in Section 2.12. A total of 360 shrimp were randomly assigned to four groups (three replicate tanks per group, with 30 shrimp per tank):

NC: Negative control (unchallenged), fed a commercial diet coated with peanut oil.
PC: Positive control (challenged), fed a commercial diet coated with peanut oil.
HKL: Challenged, fed a diet containing 64 mg/kg HKL.
Combination (Optimal): Challenged, fed a diet containing 16 mg/kg SMM-Na and 32 mg/kg HKL.

The challenge procedure, drug administration, and husbandry conditions were identical to those described in Sections 2.12.1 and 2.12.2.

#### 2.13.2 Sample collection

On days 2, 4, 6, 8, and 10 post-infection, three shrimp were randomly sampled from each tank (nine shrimp per group per time point). Hemolymph was collected using a sterile syringe and immediately transferred to pre-chilled anticoagulant tubes for hematological analysis. Hepatopancreas and intestinal tissues were aseptically dissected. Tissues were divided for subsequent analysis: one portion for bacterial load quantification, one portion snap-frozen in liquid nitrogen for RNA extraction, and one portion fixed for histopathological examination. For microbiota analysis, intestinal samples were collected on day 10 post-infection as described in Section 2.13.6.

#### 2.13.3 Hepatopancreatic bacterial load

At each sampling point, the entire hepatopancreas from one shrimp was aseptically excised and homogenized in 1 mL of sterile phosphate-buffered saline (PBS; 0.1 M, pH 7.4). The homogenate was serially diluted, and 100 µL of appropriate dilutions were spread onto TCBS agar plates. After incubation at 28 °C for 24 h, typical *V. parahaemolyticus* colonies were enumerated. Results are expressed as colony-forming units per gram of tissue (CFU/g).

#### 2.13.4 Immune parameter analysis

Total Hemocyte Count (THC): Hemolymph was diluted 1:10 with an anticoagulant solution prepared by dissolving 0.66 g EDTA-2Na, 4.56 g glucose, 3.92 g sodium chloride, and 1.588 g sodium citrate in ddH_2_O and adjusting the final volume to 200 mL (29), and 20 µL was loaded onto a hemocytometer. The total number of hemocytes was counted under a light microscope and expressed as cells × 10^6^/mL of hemolymph.

Gene expression analysis (qRT-PCR): Total RNA was extracted from hepatopancreas and intestinal tissues using RNAiso Plus reagent (Takara, Beijing, China). cDNA was synthesized from 1 µg of DNase-treated RNA using the PrimeScript™ RT Reagent Kit (Takara, Beijing, China). Quantitative PCR was performed on an ABI PRISM 7500 system (Applied Biosystems, Foster City, CA, USA) using SYBR Premix Ex Taq (Takara, Beijing, China). The relative expression of immune-related genes (*Alf, Tlr, Lec, Crustin, Lzm, CatB* in hepatopancreas; *Muc-1*, *Muc-4*, *Muc-5AC*, *Muc-19* in intestine) was normalized to *β-actin* and calculated using the 2^-ΔΔCT^ method (30). Primer sequences are listed in **Table 1**.

**Table 1:** The qPCR primers used in the present study.

Primer	Primer sequence (5′-3′)	Resources
β-actinF	AGTAGCCGCCCTGGTTGT	(46)
β-actinR	AGGATACCTCGCTTGCTCT	
Tlr-F	TGAGAGATGCCCACTGCCTG	
Tlr-R	CGCTTGAAGGTTTGTGAGGGAG	
Alf-F	TGTTCCTGGTGGCACTCTTC	
Alf-R	GTCTCCTCGTTCCTCCACAG	
Crustin-F	AACCAGAGACACCTGTTGGC	
Crustin-R	AGAATGAGGGAGGCTTGCAC	
Lzm-F	TCGAGTCGTCCTTCAACACG	
Lzm-R	AGACGTTCTTGCCGTAGTCG	
Lec-F	CGGGATCCATGAAGTTCCTAGCGCCG	
Lec-R	CGCTCGAGTATATTTCTTGAGGCAAAT	
CatB-F	CCTCTGTGGTTTTGGATGTA	
CatB-R	GATGCTGTATGCTTTGCCTC	
Muc-1-F	GGCTCGGAAGTTGGCGATGATG	(31)
Muc-1-R	CGATGGCTCAATGGCGAAGAGG	
Muc-4-F	GGAAATGACAATGACGGAAAG	
Muc-4-R	CGGGATGGTCGGGTAAG	
Muc-5AC-F	AGCAGGACTTCAACGACTACAACAG	
Muc-5AC-R	GCGCGACGCCGATGATGG	
Muc-19-F	GAAGAGGAGGAAGAGGACGAGGAG	
Muc-19-R	GGACCACCAGGCACAAGAACATC	

#### 2.13.5 Histopathological examination

Hepatopancreas and intestinal tissues were fixed in Davidson’s fixative for 24 h, transferred to 70% ethanol, and processed for paraffin embedding (32). Sections (5 µm thick) were stained with hematoxylin and eosin (H&E) and examined under an Olympus BX60 microscope (Olympus, Tokyo, Japan) to assess pathological changes. Histopathological lesions were semi-quantitatively scored on a 0–4 scale, with 0 indicating no obvious lesion and 4 indicating severe lesions.

#### 2.13.6 Intestinal microbiota analysis

Whole intestinal tracts were collected on day 10 post-infection. For each tank, intestines from three shrimp were pooled as one composite sample (n = 3 tanks per group) and immediately stored in liquid nitrogen until DNA extraction. The pooled samples included intestinal tissue together with residual luminal content. Microbial DNA was extracted using the E.Z.N.A.^®^ Soil DNA Kit (Omega Bio-tek, Norcross, GA, U.S.). The V4-V5 region of the bacterial 16S rRNA gene was amplified using the barcoded universal primers 515F (5’-GTGCCAGCMGCCGCGG-3’) and 907R (5’-CCGTCAATTCMTTTRAGTTT-3’) (33). Amplicons were pooled and subjected to paired-end sequencing (2 × 300 bp) on an Illumina NovaSeq platform. Raw sequencing data were first processed using Trimmomatic (34) for quality filtering. High-quality sequences were clustered into operational taxonomic units (OTUs) at a 97% similarity threshold using the UPARSE pipeline (35). Taxonomic classification was performed by aligning representative sequences to the SILVA (SSU138.1) 16S rRNA database (36) with an 80% confidence threshold. Alpha diversity indices (Chao1 and Shannon) were calculated using Mothur (37). Beta diversity was assessed based on Bray-Curtis distances and visualized via principal component analysis (PCA). Significant differences in microbial community structure among groups were tested using permutational multivariate analysis of variance (PERMANOVA). Differentially abundant taxa across groups were identified using linear discriminant analysis effect size (LEfSe) (38). Functional potentials of the microbiota were predicted using PICRUSt2 (39) based on the KEGG database.

### 2.14 Statistical analysis

Statistical analyses were conducted using GraphPad Prism, SPSS, and the microbiota analysis tools described above. Data are shown as mean ± SD unless otherwise indicated. Normality and homogeneity of variance were assessed before parametric analyses. Depending on the experimental design, one-way or two-way ANOVA was used, followed by Duncan’s multiple range test. Histopathological scores were analyzed using the Kruskal–Wallis test followed by Dunn’s multiple comparisons. For datasets requiring correction for multiple testing, including qPCR analyses, p values were adjusted using the Benjamini–Hochberg false discovery rate (FDR) method where appropriate. Microbiota relative abundance profiles were interpreted descriptively unless statistical tests are specifically indicated. For *in vivo* experiments, replicate tanks were treated as the independent biological units unless otherwise stated. Statistical significance was set at p < 0.05.

## 3 Results

### 3.1 *In vitro* synergistic antibacterial activity of HKL and SMM-Na

**Figure 1:**
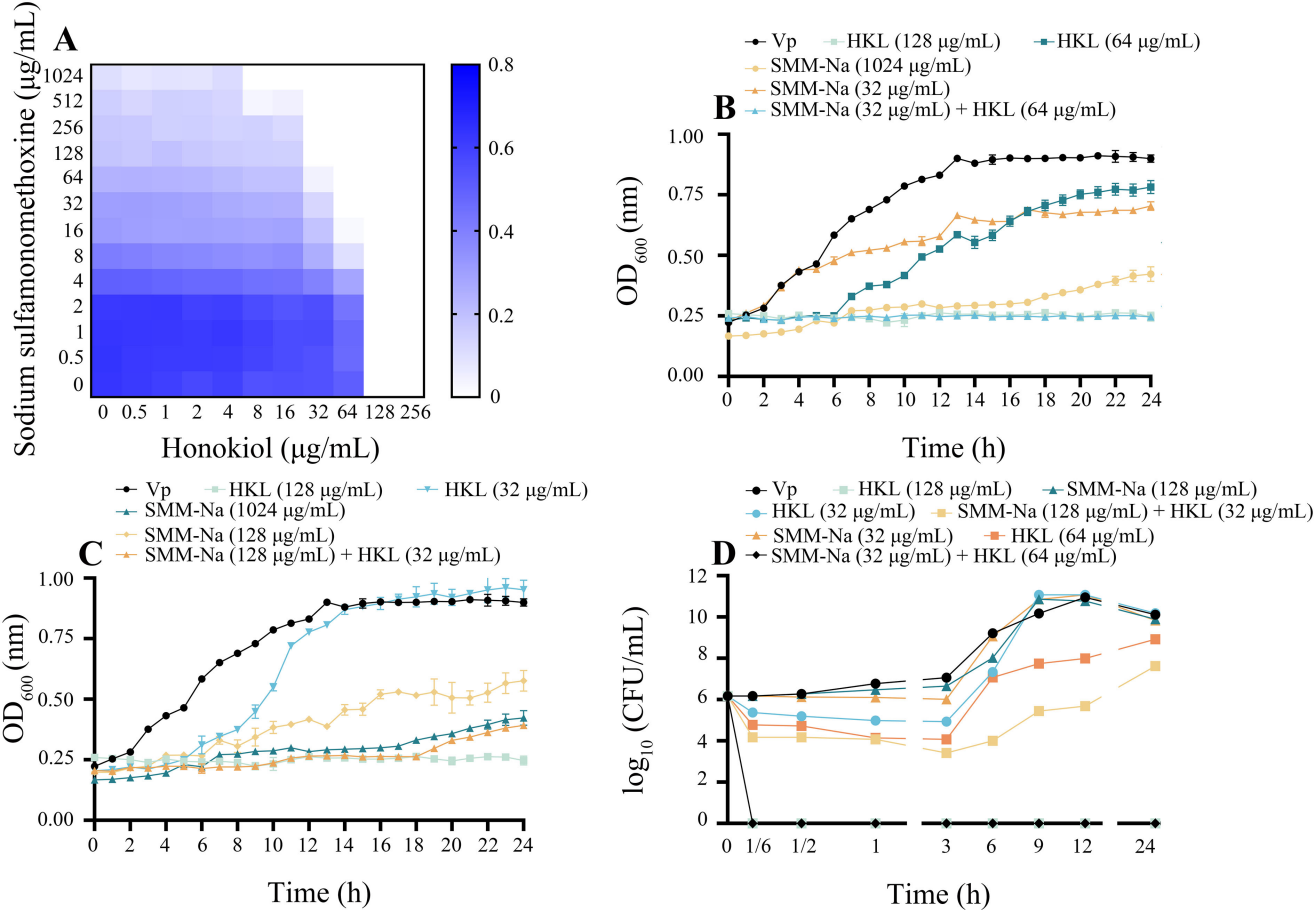
Synergistic antibacterial activity of honokiol (HKL) and sulfamonomethoxine sodium (SMM-Na) against *V. parahaemolyticus* DX190406. **(A)** Checkerboard assay illustrating the combined effects of HKL and SMM-Na, with color intensity indicating bacterial growth inhibition. The MIC of SMM-Na decreased in the presence of HKL (FICI ≤ 0.5). **(B, C)** Growth curves of *V. parahaemolyticus* DX190406 treated with HKL and SMM-Na alone or in combination at the indicated concentrations. **(D)** Time–kill kinetics following treatment with HKL and SMM-Na alone or in combination; no viable bacteria were recovered in the SMM-Na (32 μg/mL) + HKL (64 μg/mL) group from 10 min onward. Data represent at least three independent experiments and are presented as mean ± SD.

Initial antimicrobial susceptibility testing established that the MIC of HKL against *V. parahaemolyticus* DX190406 was 128 μg/mL, whereas the MIC of SMM-Na was > 1024 μg/mL. In checkerboard assays, HKL at sub-inhibitory concentrations (32-64 μg/mL) decreased the MIC of SMM-Na to 32–128 μg/mL, corresponding to an 8–32-fold reduction (**Figure 1A**). The calculated FICI values were ≤ 0.5, indicating synergy (**Figure 1A**).

This synergy was further support through bacterial growth dynamics. The combination of SMM-Na (32 μg/mL) + HKL (64 μg/mL) strongly inhibited bacterial growth throughout the observation period (**Figure 1B**). In addition, 128 μg/mL SMM-Na + 32 μg/mL HKL markedly reduced growth and prolonged the lag phase compared with the untreated control and the corresponding monotherapies (**Figure 1C**). Time-kill assays showed that HKL or SMM-Na alone produced **<** 3 log_10_ CFU/mL reductions at 24 h. In contrast, the combination SMM-Na (128 μg/mL) + HKL (32 μg/mL) achieved reductions exceeding 3 log_10_ CFU/mL at 24 h, demonstrating a bactericidal effect (**Figure 1D**). For the combination of SMM-Na (32 μg/mL) + HKL (64 μg/mL), no culturable bacteria were recovered from 10 min onward under the assay conditions, and this pattern remained unchanged through 24 h (**Figure 1D**).

### 3.2 Effects of the HKL–SMM-Na combination on biofilms

**Figure 2:**
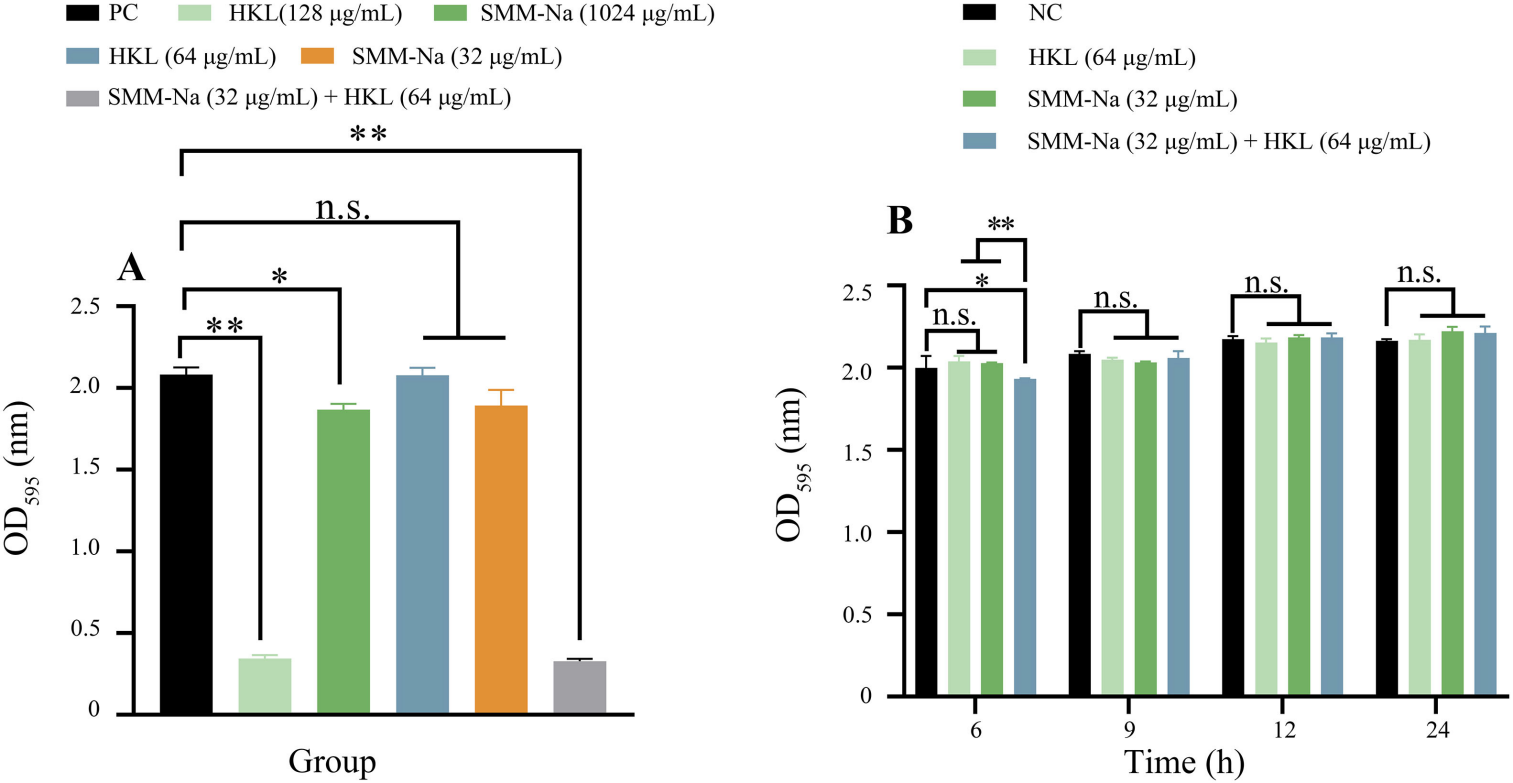
Effects of HKL and SMM-Na treatments on biofilm formation and pre-formed biofilm biomass in *V. parahaemolyticus*. **(A)** Effects of HKL, SMM-Na, or their combination on *V. parahaemolyticus* biofilm formation. Drugs were present during the biofilm-development period, and biofilm biomass was quantified by crystal violet staining. **(B)** Effects of HKL, SMM-Na, or their combination on pre-formed *V. parahaemolyticus* biofilms after different pre-formation periods. Biofilms were allowed to form for 6, 9, 12, or 24 h, washed, and then treated with HKL, SMM-Na, or the HKL–SMM-Na combination for an additional 12 h before crystal violet staining. Data are presented as mean ± SD (n = 3). Statistical significance was determined by comparison with the untreated control group within each assay or time point. *p < 0.05, **p < 0.01; n.s., not significant.

Biofilm formation was quantified by crystal violet staining (**Figure 2A**). HKL at 128 μg/mL (p < 0.01) and SMM-Na at 1024 μg/mL (p < 0.05) significantly reduced biofilm biomass compared with the untreated control (**Figure 2A**). The combination of SMM-Na (32 μg/mL) + HKL (64 μg/mL) markedly reduced biofilm biomass similar to that of HKL at 128 μg/mL (**Figure 2A**). In contrast, SMM-Na (32 μg/mL) or HKL (64 μg/mL) alone showed no comparable reduction under the tested conditions (**Figure 2A**). For pre-formed biofilms, biofilm biomass was already detected at a high level after 6 h of pre-formation and remained relatively stable from 9 to 24 h in the untreated control (**Figure 2B**). At 6 h, limited differences were observed among the treatment groups, whereas no significant reduction in biofilm biomass was observed after treatment with HKL (64 μg/mL), SMM-Na (32 μg/mL), or SMM-Na (32 μg/mL) + HKL (64 μg/mL) at 9, 12, or 24 h of biofilm pre-formation (**Figure 2B**).

### 3.3 Effects of HKL and SMM-Na on membrane permeability, morphology, ATP, and ROS

#### 3.3.1 Effects on membrane permeability and morphological alterations

**Figure 3:**
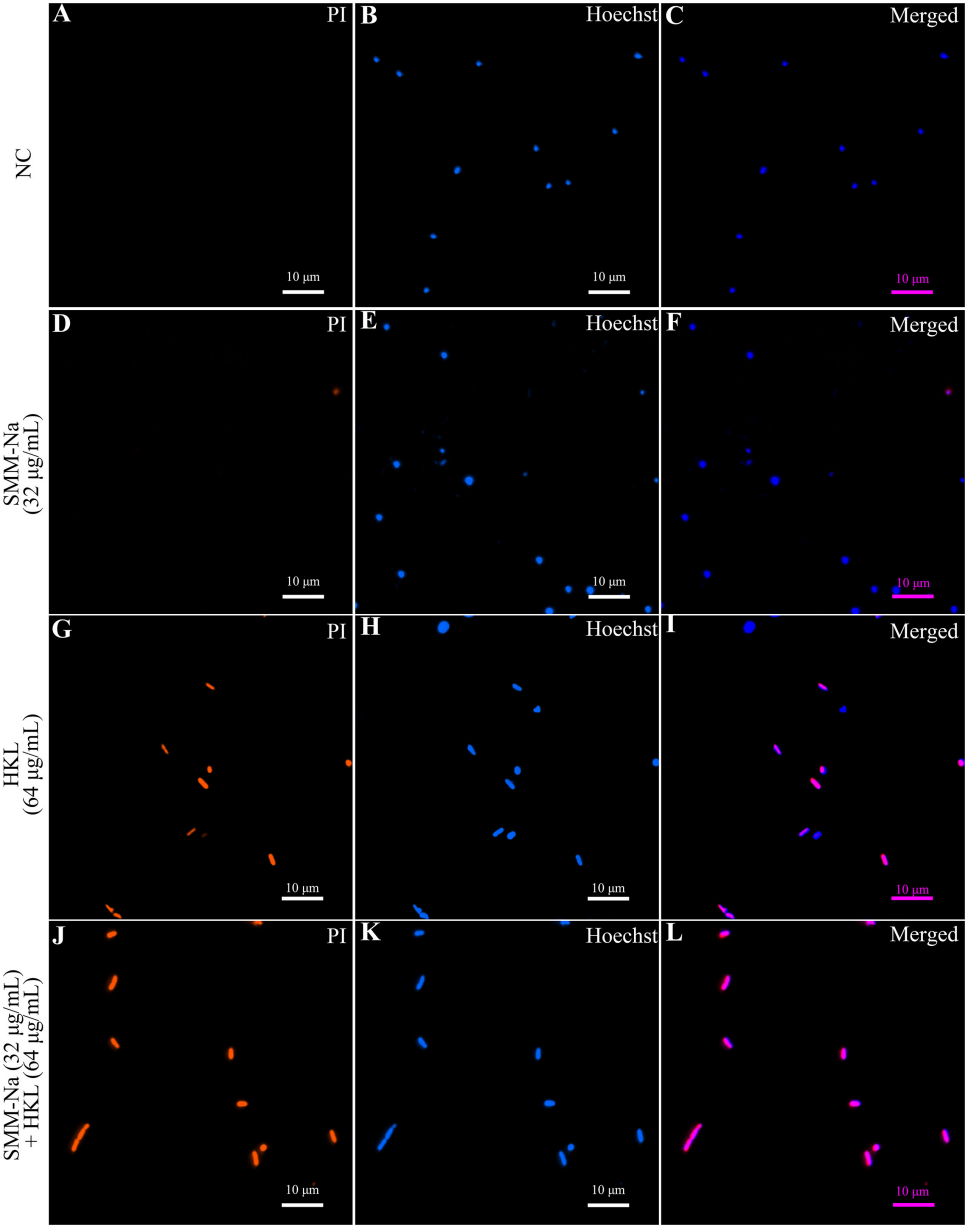
Fluorescence microscopy analysis of membrane integrity in *V. parahaemolyticus* following treatment with HKL and SMM-Na. Representative fluorescence images showing propidium iodide (PI, red), Hoechst (blue), and merged signals in bacteria from the negative control **(A–C)**, SMM-Na (32 μg/mL)–treated group **(D–F)**, HKL (64 μg/mL)–treated group **(G–I)**, and the combined SMM-Na (32 μg/mL) + HKL (64 μg/mL) treatment group **(J–L)**. Increased PI uptake indicates compromised cytoplasmic membrane integrity. Scale bars, 20 μm.

Fluorescence staining with Hoechst 33342 and propidium iodide (PI) revealed pronounced differences in membrane integrity among the treatment groups. As shown in **Figure 3**, bacteria in the negative control and SMM-Na–treated groups exhibited predominantly Hoechst-positive and minimal PI signal. Treatment with HKL alone increased the number of PI-positive cells. In contrast, SMM-Na (32 μg/mL) + HKL (64 μg/mL) resulted in a stronger PI signal and more PI-positive cells in merged images (**Figure 3**).

**Figure 4:**
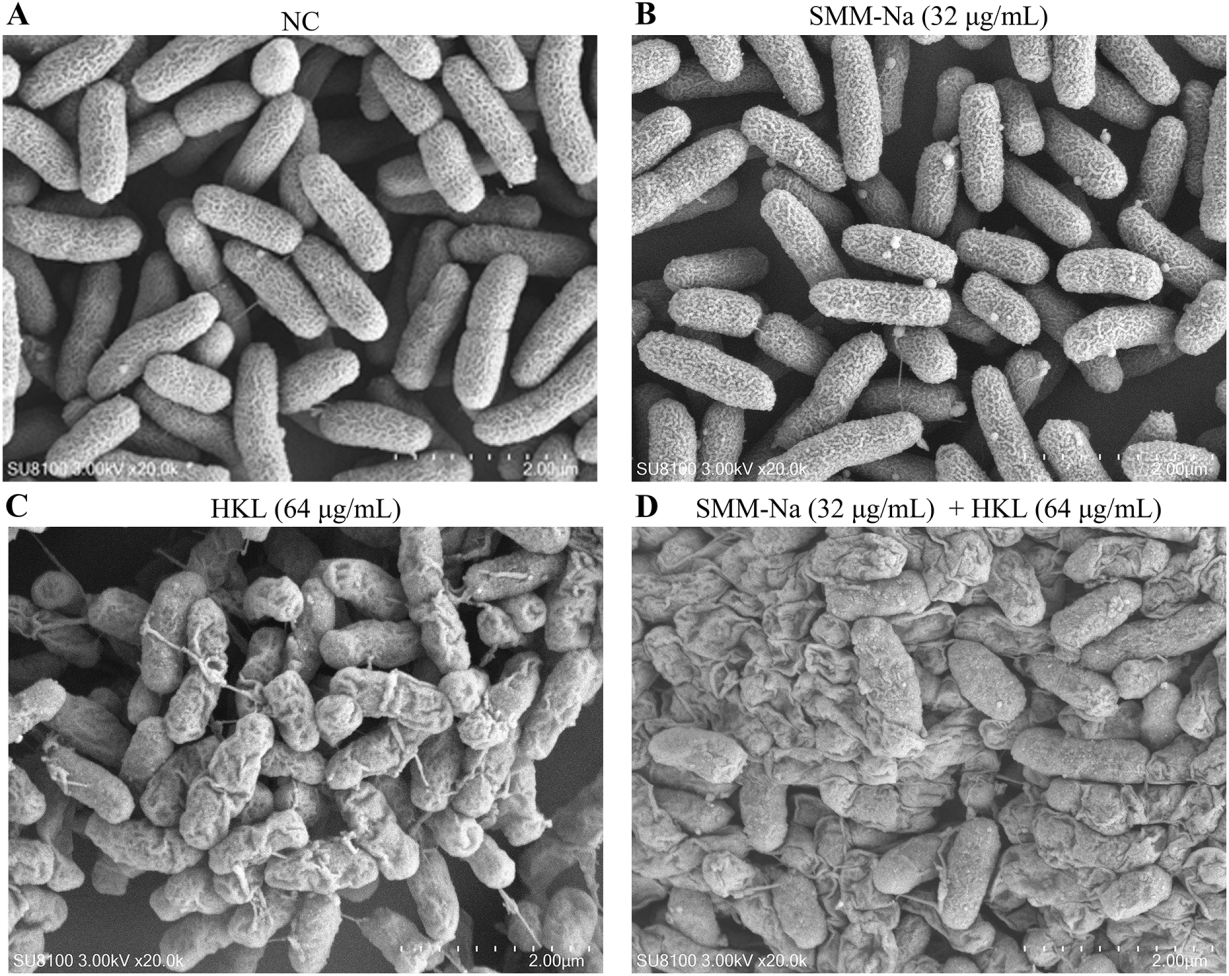
Scanning electron microscopy (SEM) analysis of morphological alterations in *V. parahaemolyticus* induced by HKL and SMM-Na. Representative SEM images of bacteria from the negative control **(A)**, SMM-Na (32 μg/mL) group **(B)**, HKL (64 μg/mL) group **(C)**, and the combined SMM-Na (32 μg/mL) + HKL (64 μg/mL) group **(D)**. Scale bars, 2 μm; magnification, 20,000×.

SEM revealed corresponding morphological differences. As shown in **Figure 4**, the negative control and SMM-Na–treated bacteria maintained a typical rod-shaped morphology with smooth and intact cell surfaces. HKL treatment alone induced moderate surface irregularities and localized deformation. Notably, bacteria exposed to the combination exhibited extensive morphological damage, characterized by cell surface collapse, pronounced wrinkling, and disruption of overall cellular architecture, consistent with substantial membrane and structural destabilization.

#### 3.3.2 Effects on intracellular ATP

**Figure 5:**
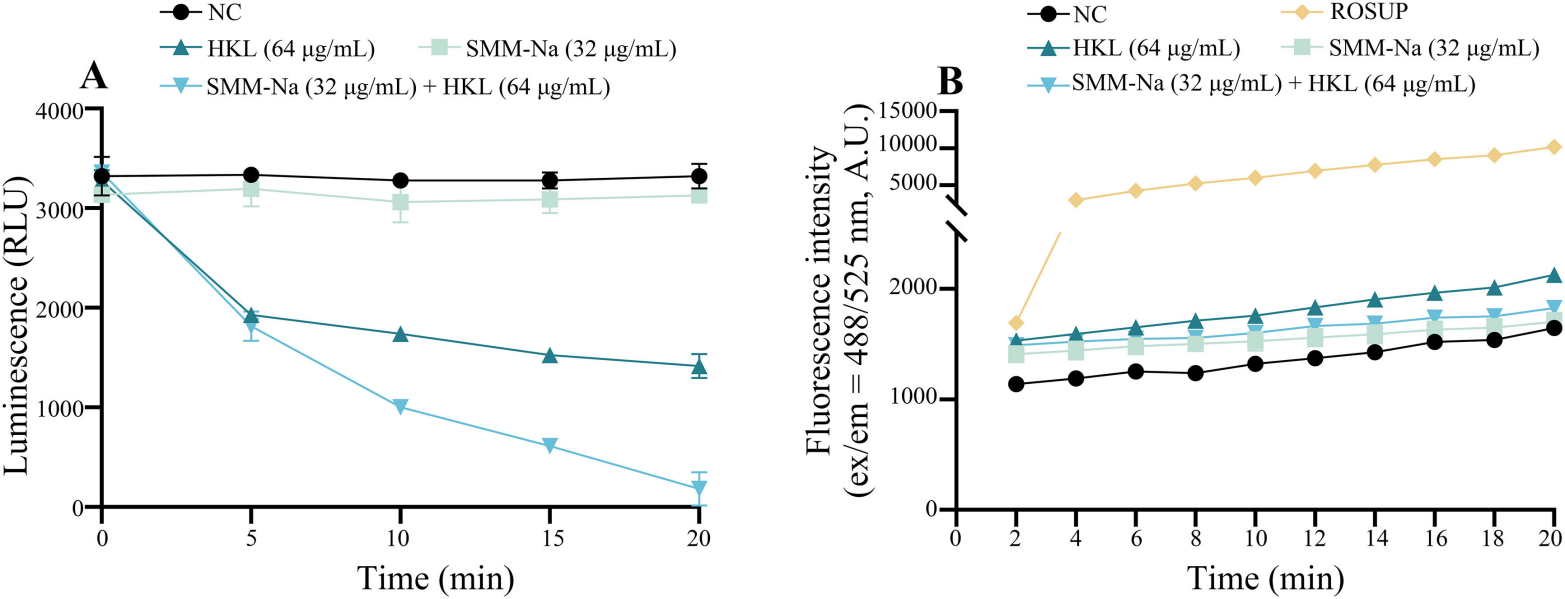
Effects of HKL and SMM-Na treatments on intracellular ATP and ROS levels in *V. parahaemolyticus*. **(A)** Time-course of intracellular ATP levels within 20 min after treatment, measured by luminescence. **(B)** Time-course of intracellular ROS levels within 20 min after treatment, measured using DCFH-DA fluorescence (Ex 488 nm/Em 525 nm); ROSUP served as the positive control. Data are presented as mean ± SD (n = 3).

Intracellular ATP levels remained relatively stable in the SMM-Na (32 μg/mL) group over the 20-min period, whereas HKL (64 μg/mL) led to a progressive decline (**Figure 5A**). The combination SMM-Na (32 μg/mL) + HKL (64 μg/mL) showed a more pronounced decrease, reaching the lowest ATP levels at the later time points (**Figure 5A**).

#### 3.3.3 Effects on ROS levels

DCFH-DA fluorescence increased over time in all groups, with ROSUP showing a marked increase as a positive control (**Figure 5B**). Compared with the negative control, HKL (64 μg/mL) and the combination groups exhibited a higher fluorescence signal over the measurement period, while the combination did not show a higher signal than HKL alone (**Figure 5B**). Together, these assays showed that the HKL–SMM-Na combination was accompanied by increased membrane permeability, marked morphological damage, and rapid depletion of intracellular ATP (**Figures 3**–**5**).

### 3.4 Protective effect of the low-dose SMM-Na and HKL combination in *P. vannamei*

#### 3.4.1 Survival rate

**Figure 6:**
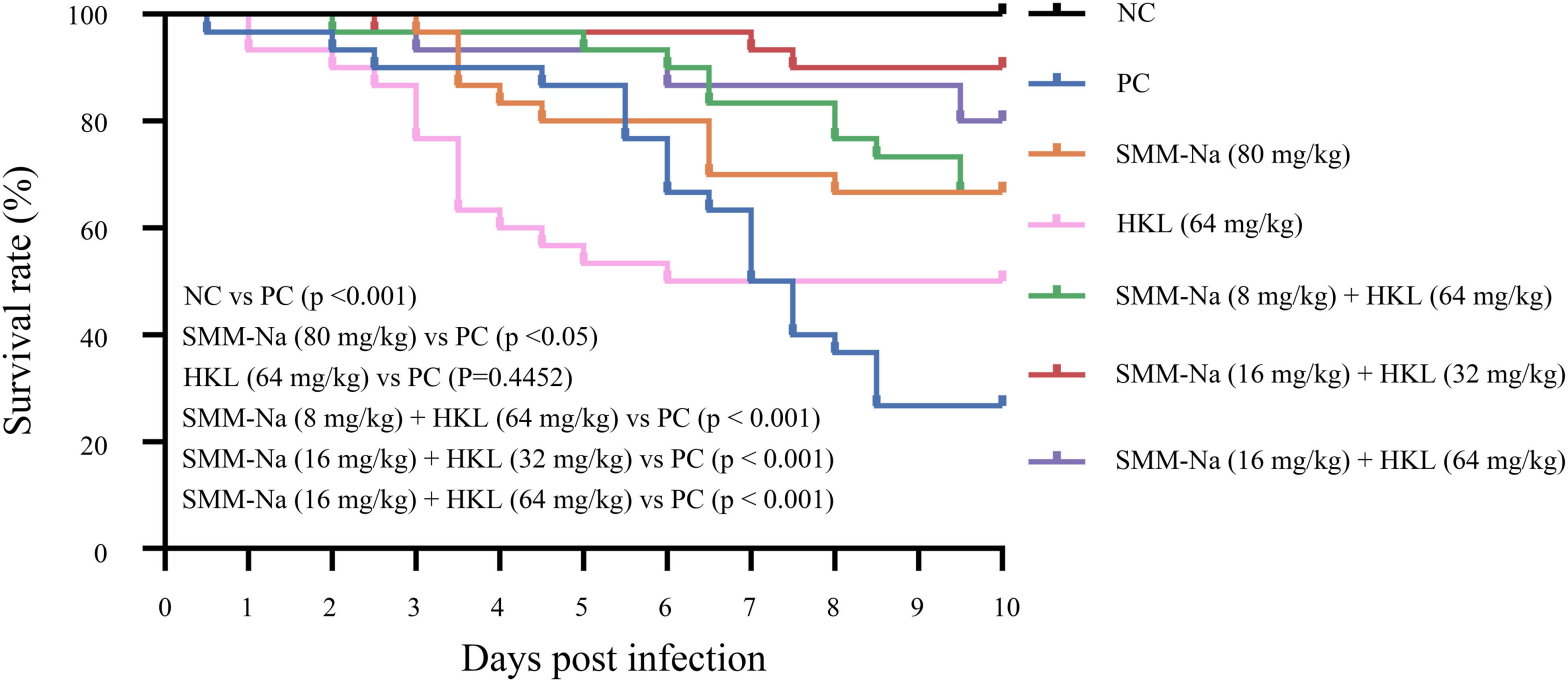
Survival curves of *P. vannamei* following challenge with *V. parahaemolyticus*. Shrimp were challenged with *V. parahaemolyticus* and fed diets supplemented with SMM-Na, HKL, or their combinations at the indicated doses. Survival was monitored daily for 10 days post-infection.

In the immersion challenge model, survival in the positive control (PC) group decreased to 26.7% at day 10 post-infection, whereas the SMM-Na monotherapy (80 mg/kg) group and the HKL (64 mg/kg) group achieved survival rates of 66.7% and 50%, respectively (**Figure 6**). In contrast, shrimp receiving the SMM-Na (16 mg/kg) + HKL (32 mg/kg) diet showed the highest survival, reaching 86.6%, and the other two combination groups, namely SMM-Na (8 mg/kg) + HKL (64 mg/kg) and SMM-Na (16 mg/kg) + HKL (64 mg/kg), achieved survival rates of 66.7% and 80%. The survival curves indicated that mortality in the PC group increased markedly after approximately day 5–6, while mortality was reduced in the treatment groups, particularly in the combination group (**Figure 6**). Based on the survival-based dose-finding screening, the SMM-Na (16 mg/kg) + HKL (32 mg/kg) diet was selected for subsequent efficacy assessments.

#### 3.4.2 Effects on hepatopancreatic bacterial load and hemocyte responses

**Figure 7:**
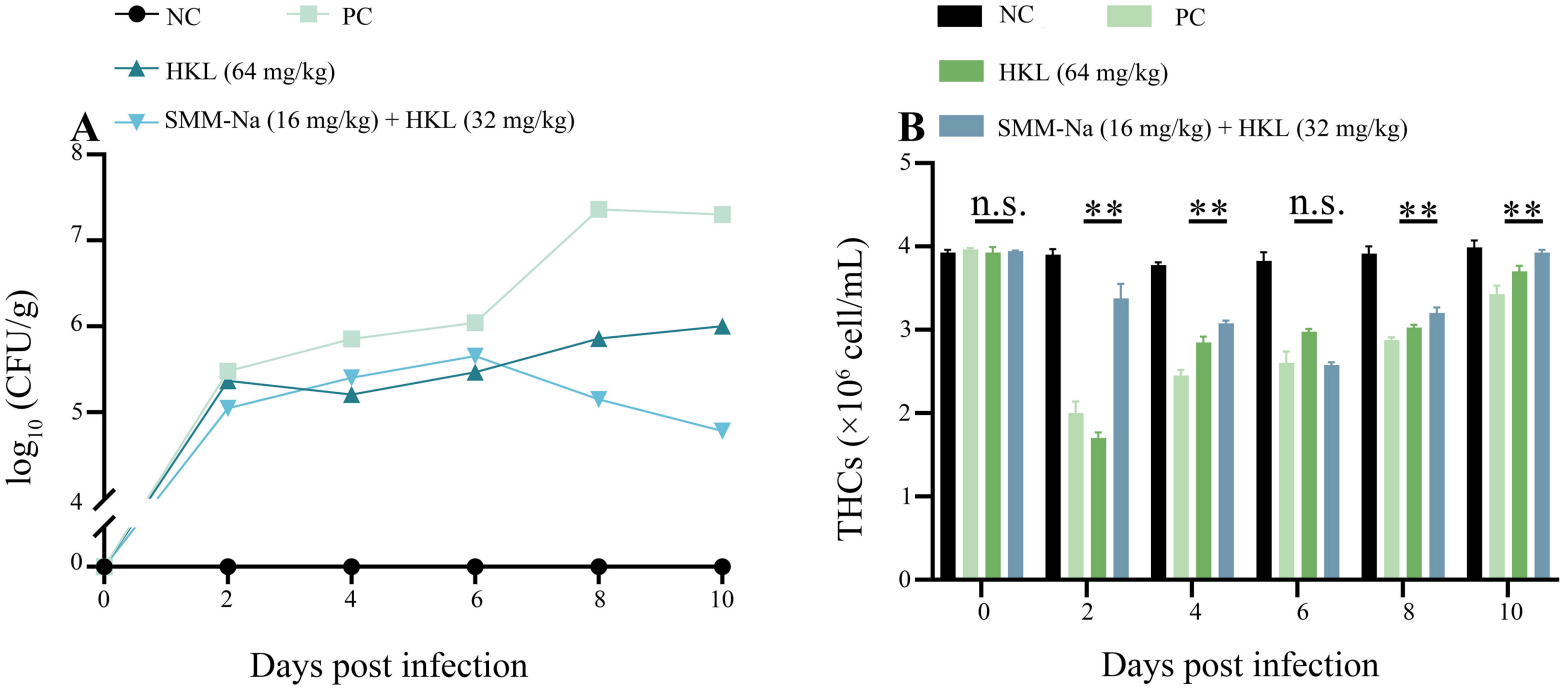
Hepatopancreatic bacterial load **(A)** and total hemocyte count (THC) **(B)** of *P. vannamei* at different time points following *V. parahaemolyticus* challenge. Shrimp were orally administered diets supplemented with HKL, SMM-Na, or their combination. **p < 0.01 compared with the negative control (NC; uninfected and untreated shrimp) group; n.s., not significant.

Hepatopancreatic bacterial loads were quantified over the infection course (**Figure 7A**). The PC group showed a progressive increase in bacterial burden, reaching the highest levels at later time points. HKL (64 mg/kg) reduced hepatopancreatic bacterial counts relative to the PC group across the sampling period. Notably, by day 10, bacterial loads in the combination group decreased to below 10^5^ CFU/g, whereas the PC group exceeded 10^7^ CFU/g, corresponding to a reduction of more than two orders of magnitude (**Figure 7A**).

THC decreased sharply in the PC group by day 2 post-infection and remained below the NC level thereafter (**Figure 7B**). In the HKL group, THC also declined at day 2 but showed partial recovery over time. In contrast, the combination group maintained higher THC levels than the PC group at most time points and approached the NC level by the end of the trial (**Figure 7B**).

#### 3.4.3 Immune-related gene expression

**Figure 8:**
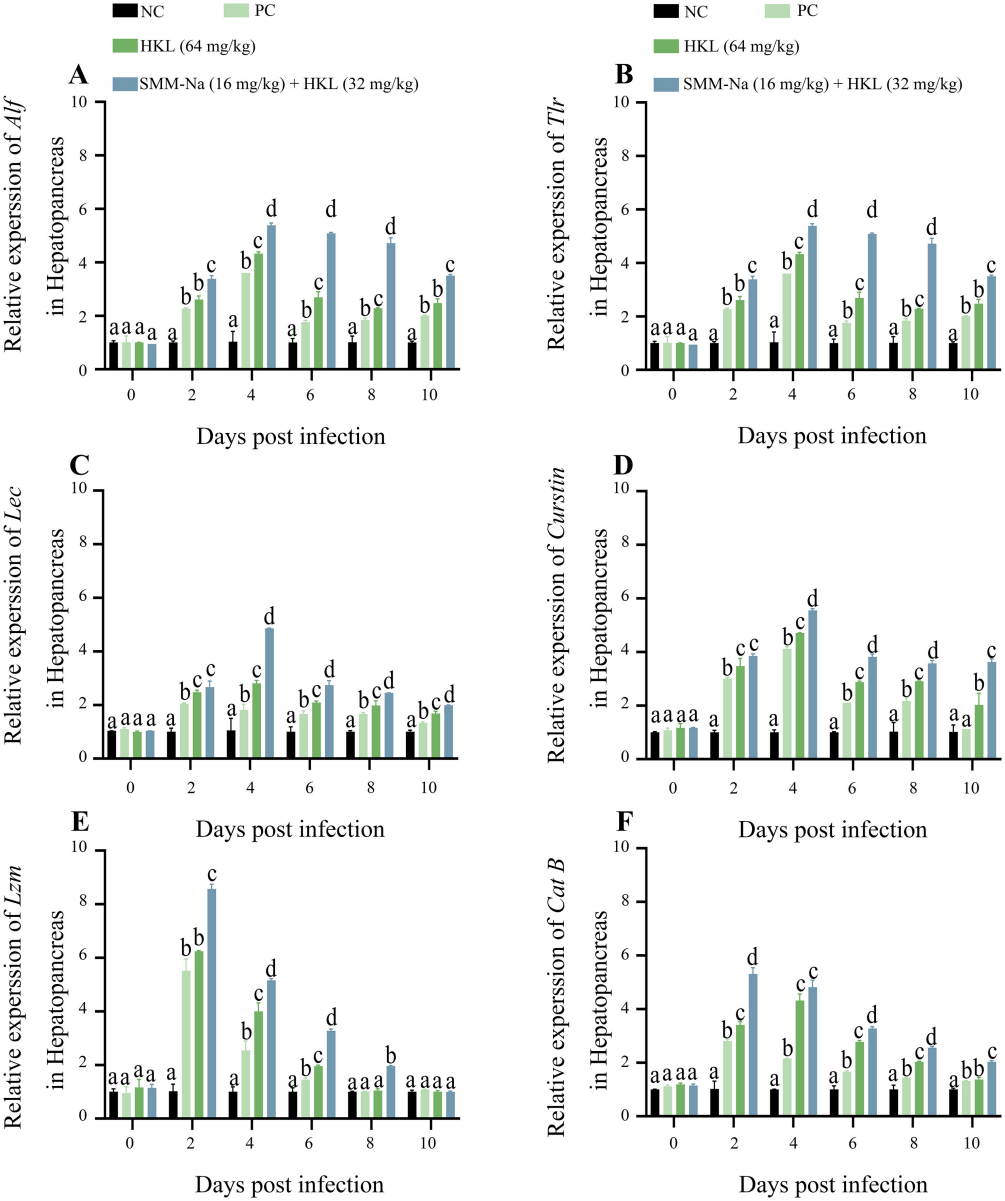
Profiles of mRNA expression levels of immunity-related factors *Alf* **(A)**, *Tlr* **(B)**, *Lec* **(C)**, *Crustin* **(D)**, *Lzm* **(E)**, and *CatB* **(F)** in the hepatopancreas of *P. vannamei* after oral administration. Significant differences among different groups at the same time point are indicated by different letters (p < 0.05).

**Figure 9:**
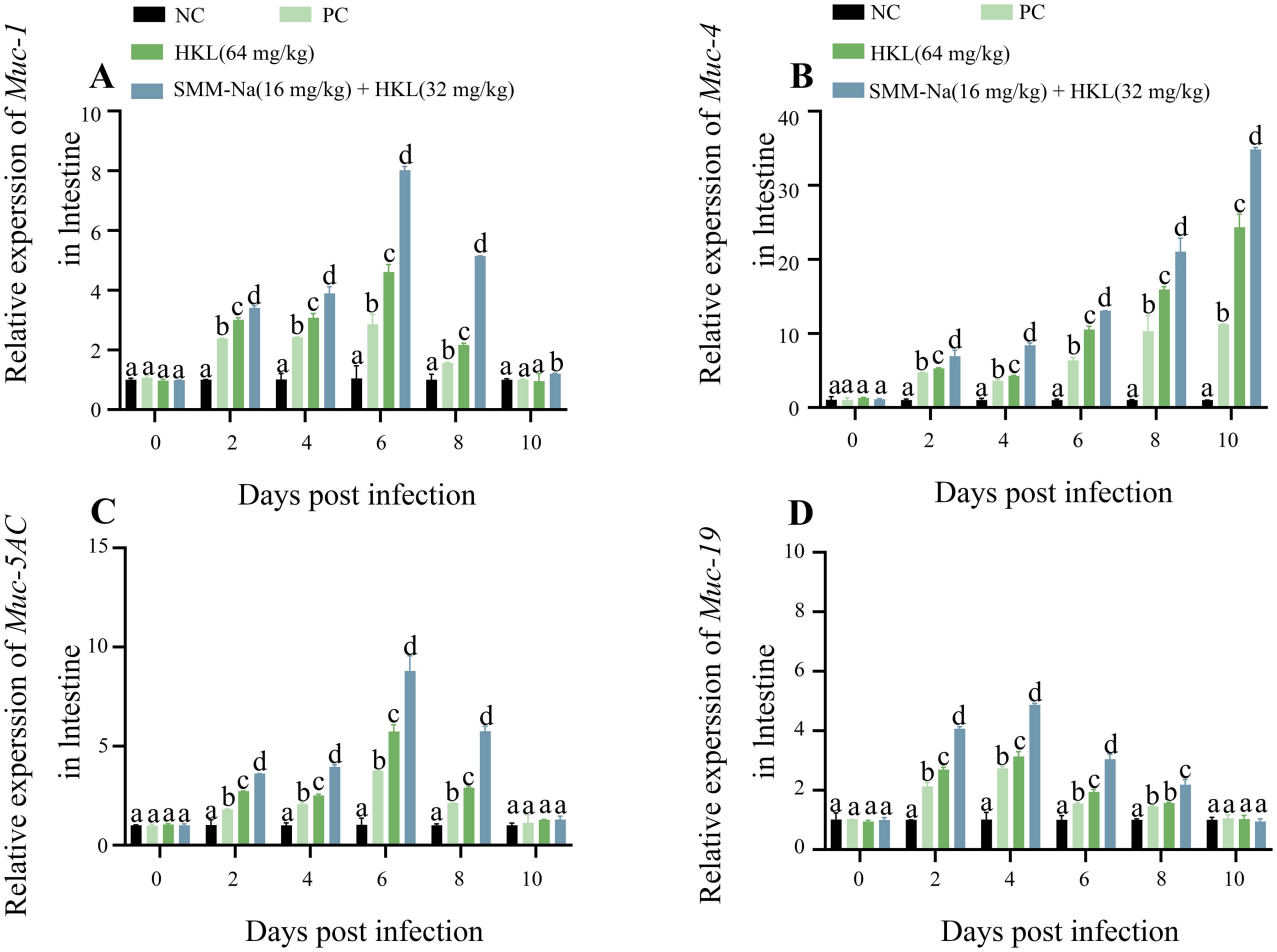
Profiles of mRNA expression levels of immunity-related factors *Muc-1* **(A)**, *Muc-4* **(B)**, *Muc-5AC* **(C)**, and *Muc-19* **(D)** in the intestine of *P. vannamei* after oral administration. Significant differences among different groups at the same time point are indicated by different letters (p < 0.05).

The expression of immune-related genes was evaluated in hepatopancreas and intestine (**Figures 8**, **9**). In the hepatopancreas, transcripts associated with pathogen recognition and effector responses (*Alf*, *Tlr*, *Lec*, *Crustin*, *Lzm*, and *CatB*) showed time-dependent induction after infection, with generally higher expression in the combination group than in the PC group at multiple time points (**Figures 8A–F**). HKL alone also increased the expression of several genes relative to the PC group at selected time points, although the magnitude was typically lower than that observed in the combination group (**Figure 8**).

In the intestine, mucosal barrier–related genes (*Muc-1*, *Muc-4*, *Muc-5AC*, and *Muc-19*) displayed time-dependent upregulation, and the combination group showed higher expression than the PC group at multiple time points (**Figures 9A–D**).

#### 3.4.4 Histopathology of the hepatopancreas and intestine

**Figure 10:**
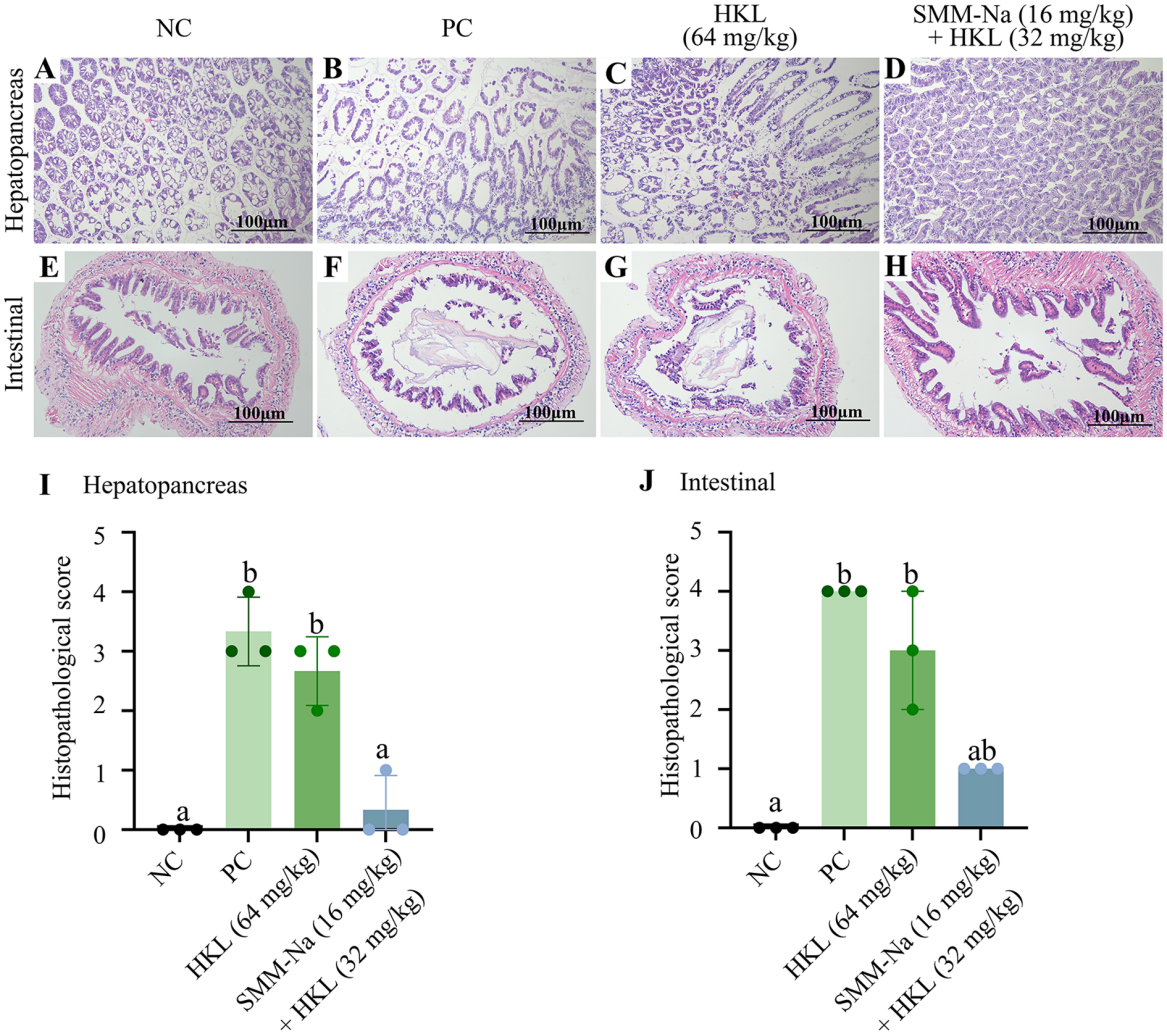
Histological analysis of the hepatopancreas and intestine of *P. vannamei* following *V. parahaemolyticus* challenge. Hepatopancreatic sections: negative control **(A)**, positive control **(B)**, HKL (64 mg/kg) **(C)**, and SMM-Na (16 mg/kg) + HKL (32 mg/kg) **(D)**. Intestinal sections: negative control **(E)**, positive control **(F)**, HKL (64 mg/kg) **(G)**, and SMM-Na (16 mg/kg) + HKL (32 mg/kg) **(H)**. **(I)** Semi-quantitative histopathological scores of the hepatopancreas. **(J)** Semi-quantitative histopathological scores of the intestine. Data are presented as mean ± SD (n = 3). Different letters indicate significant differences among groups (p < 0.05). Scale bars = 100 μm.

Histopathological analysis revealed severe tissue damage in the hepatopancreas and intestine of *V. parahaemolyticus*-infected shrimp (PC group). The hepatopancreatic tubules exhibited extensive epithelial cell sloughing, luminal expansion, and significant necrosis. In the intestine, disruption of the mucosal epithelium and structural integrity was prominent (**Figures 10A, B, E, F**). In contrast, shrimp treated with the SMM-Na + HKL combination showed markedly attenuated histopathological lesions. In the combination group, the architecture of the hepatopancreatic tubules was largely preserved, with only focal and mild epithelial detachment. The intestinal mucosa also maintained a more intact structure with fewer apparent necrotic changes compared to the PC group (**Figure 10**). The hepatopancreatic lesion scores were 0.00 ± 0.00, 3.33 ± 0.58, 2.67 ± 0.58, and 0.33 ± 0.58 in the NC, PC, HKL, and SMM-Na + HKL groups, respectively (**Figure 10I**; **Supplementary Table 1**). The intestinal lesion scores were 0.00 ± 0.00, 4.00 ± 0.00, 3.00 ± 1.00, and 1.00 ± 0.00 in the NC, PC, HKL, and SMM-Na + HKL groups, respectively (**Figure 10J**; **Supplementary Table 1**). Representative replicate H&E images used for scoring are provided in **Supplementary Figures S1** and **S2**.

#### 3.4.5 Gut-associated microbiota composition and predicted functional profiles

**Figure 11:**
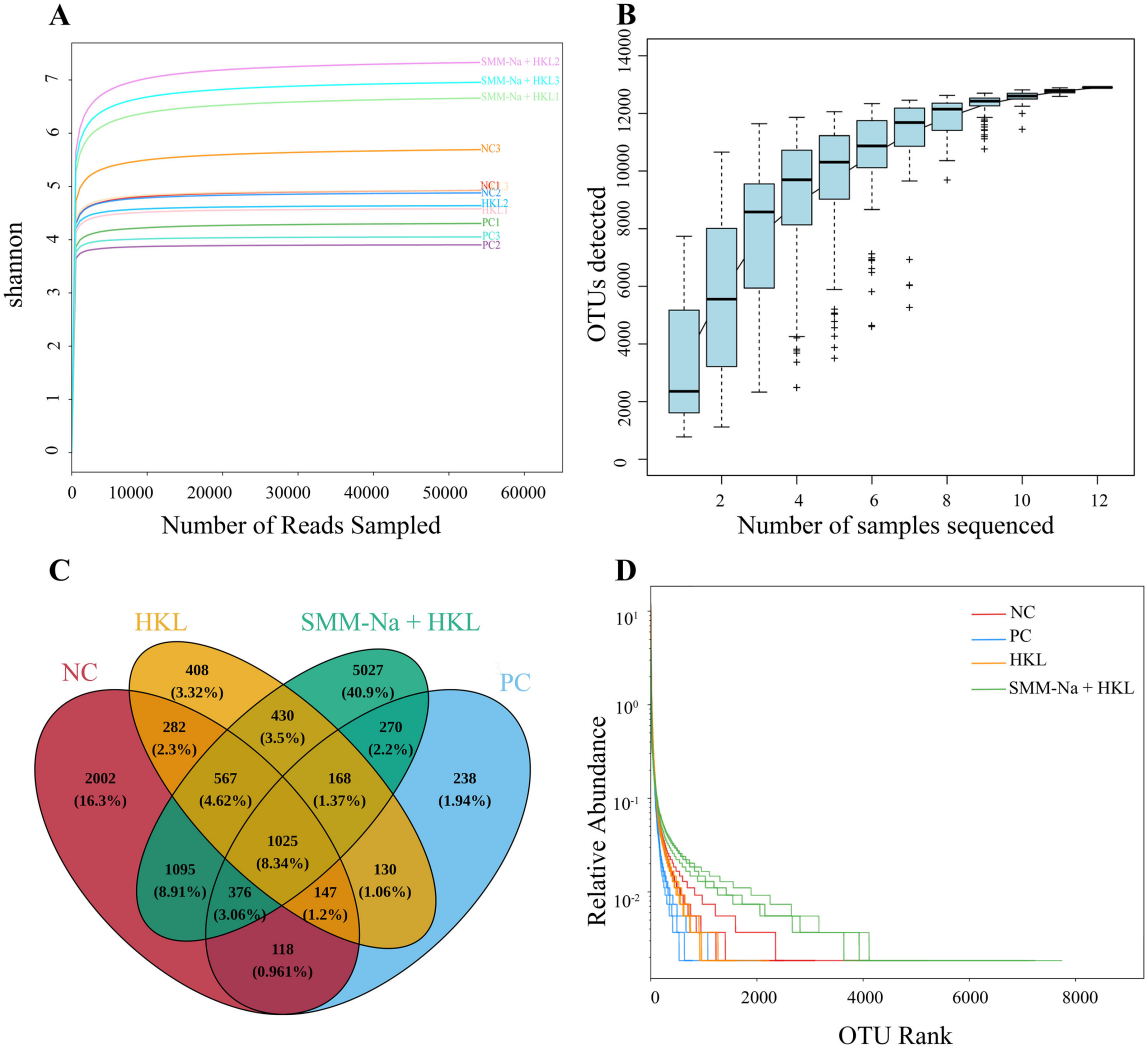
Sequencing depth and diversity summaries of gut-associated microbiota in *P. vannamei* (day 10 post-infection). **(A)** Rarefaction curves based on the Shannon–Wiener index. **(B)** Species accumulation curves. **(C)** Venn diagram of observed OTUs. **(D)** Rank–abundance curves. Samples were pooled (three shrimp per tank) with three biological replicates per group.

16S rRNA gene sequencing was performed on day 10 gut-associated samples (**Figure 11**; **Table 2**). Rarefaction analysis indicated sufficient sequencing depth for downstream analyses (**Figure 11A**). Alpha diversity indices differed significantly among groups (**Table 2**). Compared with the NC group, the PC group showed significantly lower richness and diversity, with reduced Observed species, Chao1, ACE, Shannon, and Simpson indices (p < 0.05). The HKL group showed mixed patterns: Chao1 and Simpson were comparable to the NC group, whereas Observed species and ACE remained at levels similar to the PC group; the Shannon index was intermediate between the NC and PC groups (**Table 2**). By contrast, the SMM-Na + HKL group displayed the highest values across all five indices (Observed species, Chao1, ACE, Shannon, and Simpson), which were significantly higher than those of the NC and PC groups (p < 0.05) (**Table 2**).

**Table 2:** Alpha diversity indices of intestine microbiota (n=3, mean ± SD).

Group	Observed species index	Richness assessment		Diversity assessment	
		Chao1	ACE	Shannon	Simpson
NC	3500.33 ± 1097.48^b^	6863.12 ± 2324.54^b^	7092.86 ± 2192.55^b^	5.15 ± 0.37^b^	0.9748 ± 0.0040^b^
PC	1242 ± 566.33^c^	1807.97 ± 994.54^c^	1949.79 ± 1169.60^c^	4.08 ± 0.16^c^	0.9551 ± 0.0028^c^
HKL	1744.66 ± 320.89^c^	2740.89 ± 526.40^b^	2756.57 ± 628.36^c^	4.71 ± 0.15^bc^	0.9701 ± 0.0030^b^
SMM-Na + HKL	6964 ± 248.27^a^	12629.21 ± 1185.39^a^	11641.15 ± 644.30^a^	6.92 ± 0.27^a^	0.9931 ± 0.0023^a^

Values with significant differences in the same column are marked with different lowercase letters (p < 0.05).

**Figure 12:**
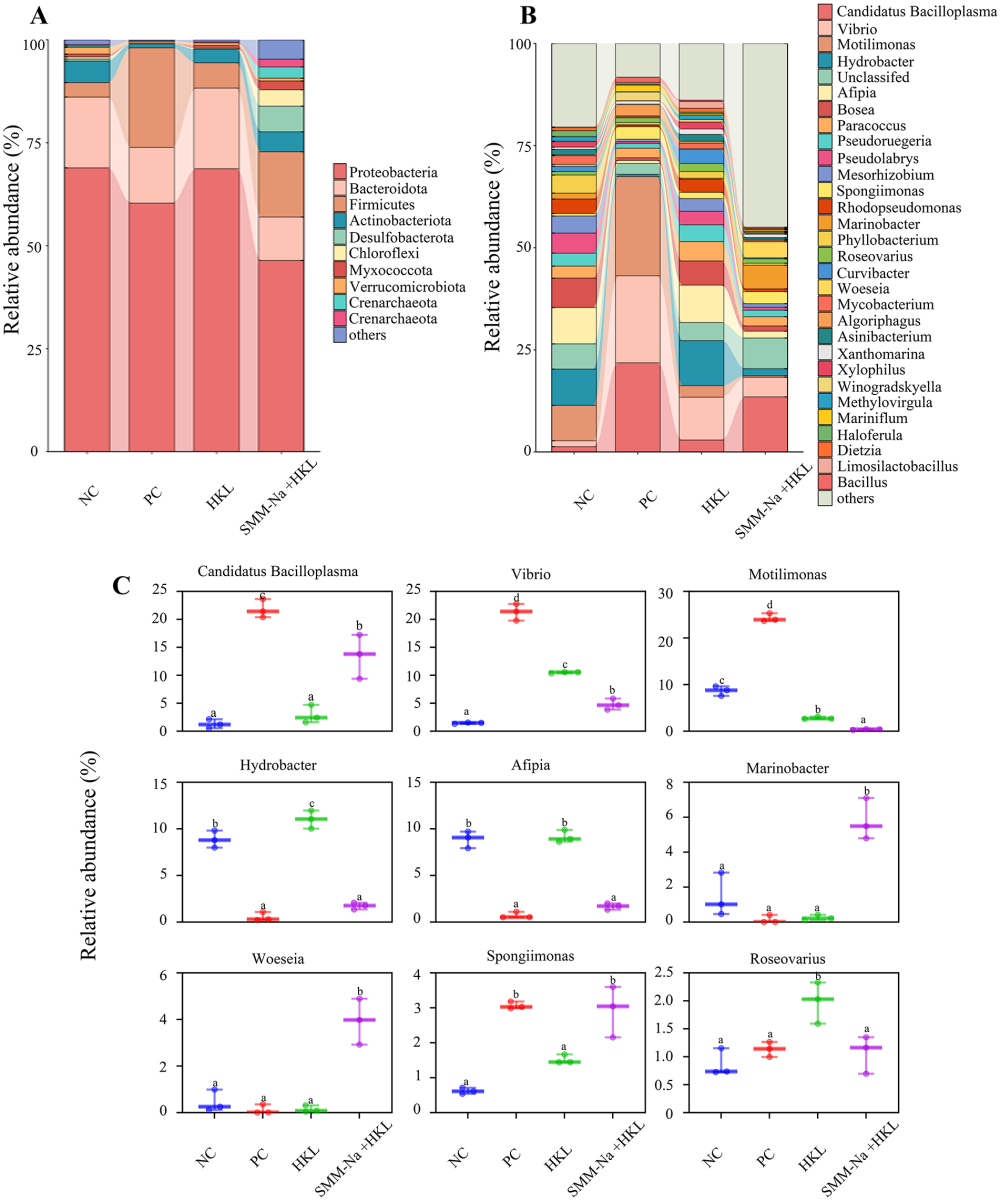
Composition and relative abundance of gut-associated microbiota in *P. vannamei* (day 10 post-infection). **(A)** Relative abundance at the phylum level. **(B)** Relative abundance at the genus level. **(C)** Relative abundance of selected genera. Data are presented as mean ± SD (n = 3). Different letters indicate significant differences among groups (p < 0.05).

Principal component analysis (PCA) based on Bray–Curtis distances showed clear separation of microbial communities among groups (**Figure 12**). At the phylum level, Proteobacteria dominated across groups, while relative abundances of several major phyla differed among treatments (**Figure 12A**). At the genus level, the PC group exhibited a significantly higher relative abundance of *Vibrio* than the other groups (p < 0.05), whereas *Vibrio* abundance was significantly reduced in the HKL and SMM-Na + HKL groups (**Figures 12B, C**). For *Motilimonas*, the PC group also showed the highest abundance, and the HKL and SMM-Na + HKL groups displayed significantly lower levels (p < 0.05), with the lowest abundance observed in the SMM-Na + HKL group (**Figure 12C**). In contrast, *Roseovarius* abundance was significantly higher in the HKL group than in the NC, PC, and SMM-Na + HKL groups (p < 0.05), while no significant differences were observed among the latter three groups (**Figure 12C**).

**Figure 13:**
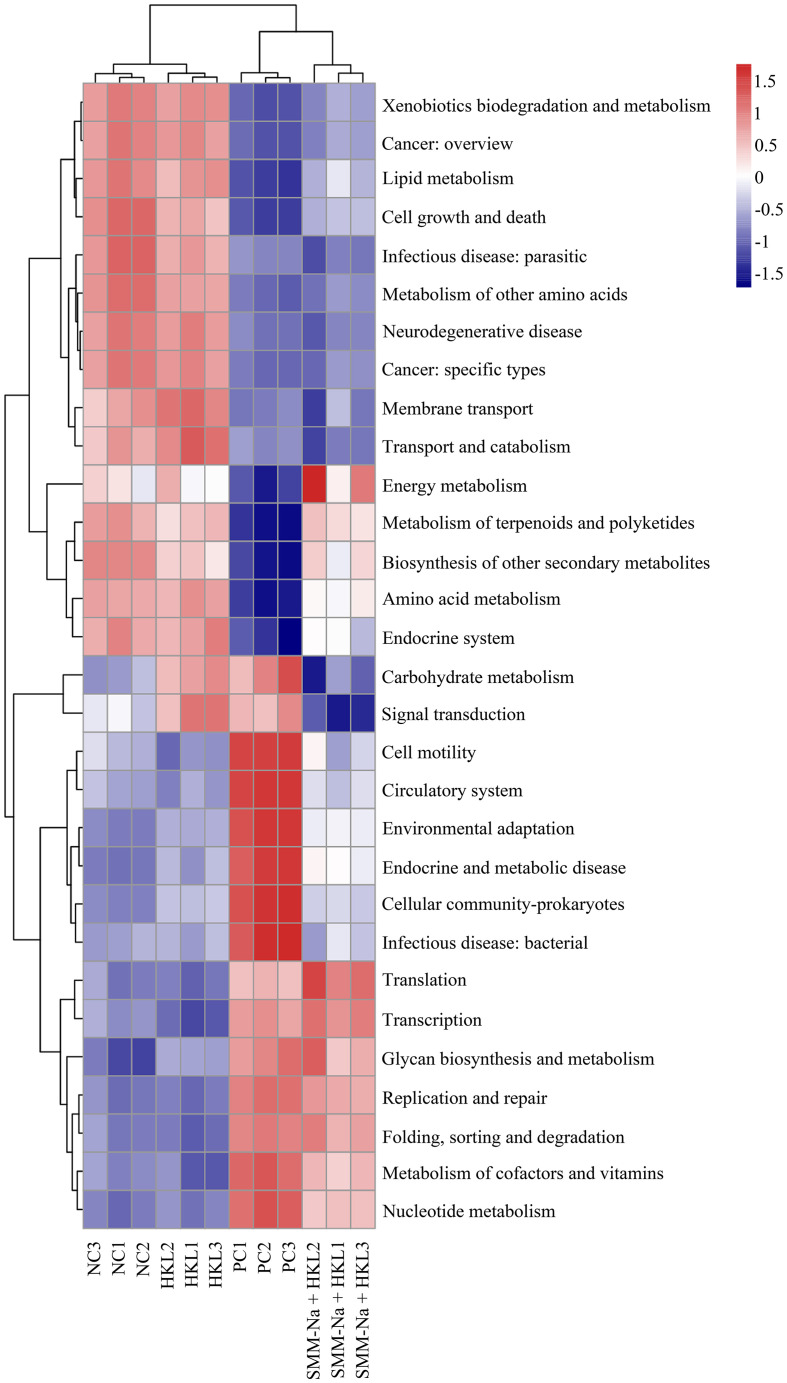
Heatmap of predicted functional profiles of gut-associated microbiota based on KEGG level-2 categories (day 10 post-infection). Heatmap showing the top 30 predicted functions (KEGG level-2). Values are Z-score–scaled across samples, and hierarchical clustering was performed for both samples and functions (n = 3 per group).

Functional prediction based on KEGG level-2 categories revealed group-dependent differences in predicted pathway profiles (**Figure 13**). Hierarchical clustering showed that samples from the same group tended to cluster together. Relative to the NC group, the PC group exhibited higher predicted abundances in categories including cell motility, signal transduction, and infectious disease: bacterial, whereas several metabolism-related categories (e.g., amino acid metabolism and xenobiotics biodegradation and metabolism) showed lower predicted abundances (**Figure 13**). In the HKL and SMM-Na + HKL groups, multiple categories displayed predicted abundances distinct from those in the PC group, with pathway-specific patterns observed across metabolic and disease-associated functions (**Figure 13**).

## 4 Discussion

The present study was motivated by the need to improve therapeutic options for AHPND while reducing the amount of antibiotic required for effective bacterial control in shrimp aquaculture. By integrating *in vitro* antibacterial assays, an *in vivo* infection model, and intestinal microbiota analysis, this work demonstrates that honokiol (HKL), a plant-derived lignan, functions as a natural-origin potentiator of SMM-Na against AHPND-causing *V. parahaemolyticus*. *In vitro*, sub-inhibitory concentrations of HKL synergistically enhanced the bactericidal activity of SMM-Na, an effect associated with bacterial membrane disruption, ATP depletion, and inhibition of biofilm formation. *In vivo*, the dietary combination of low-dose SMM-Na (16 mg/kg) and HKL (32 mg/kg) significantly improved shrimp survival, reduced hepatopancreatic bacterial load, attenuated infection-associated hemocyte depletion and tissue damage, and upregulated key immune and mucosal barrier genes. Furthermore, the combined treatment was associated with changes in the intestinal microbiota, including increased alpha diversity, reduced relative abundance of *Vibrio*, and altered predicted functional profiles. Together, these results support HKL-assisted SMM-Na treatment as a dose-sparing combination strategy for improving the control of AHPND-associated *Vibrio* infection, particularly in the context of reduced susceptibility to conventional antibiotics and the need to limit therapeutic antimicrobial input in aquaculture.

### 4.1 *In vitro* synergy is driven by membrane-associated physiological disruption

The observed *in vitro* synergy between HKL and SMM-Na is most consistently explained by a mechanism centered on bacterial membrane disruption and consequent physiological collapse, rather than by the inhibition of a specific molecular target. Checkerboard and time-kill assays demonstrated that sub-MIC concentrations of HKL drastically reduced the MIC of SMM-Na and achieved bactericidal effects. This aligns with the recognized principle that the outer membrane of Gram-negative bacteria is a major barrier to antibiotics, and its compromise is a common strategy for antibiotic adjuvants (40). Our direct evidence—increased propidium iodide uptake, obvious morphological alterations observed via SEM, and rapid ATP depletion—collectively points to disruption of membrane integrity and energy homeostasis. Consistently, the HKL–SMM-Na combination inhibited biofilm formation, whereas its effect on pre-formed biofilm biomass was limited under the tested conditions. In the ROS assay, HKL-containing treatments increased DCFH-DA fluorescence compared with the untreated control, whereas the combination showed a fluorescence pattern similar to HKL alone during the early detection period. Together with the membrane permeability, SEM, and ATP data, this result suggests that membrane perturbation and energy depletion were the dominant early physiological changes associated with HKL-mediated potentiation of SMM-Na (7). This physiology-centered interpretation is supported by quantitative evidence from antibiotic-enhancing combinations, particularly those linked to bacterial envelope perturbation. For example, oxethazine–colistin and naringenin–colistin combinations produced synergistic activity against Gram-negative bacteria, with FICI values in the synergistic range and >2 and >3 log10 reductions, respectively, in time-kill assays (8, 41). Domiphen bromide reduced colistin MICs by 4–1024-fold in colistin-resistant Gram-negative bacteria and decreased biofilm formation by approximately twofold in strongly biofilm-forming strains (42). In aquaculture, 50 mg/L oxolinic acid combined with 50 mg/L oxytetracycline produced a larger inhibition zone against *V. parahaemolyticus* than either agent alone (20.15 ± 1.15 mm versus 10.60 ± 0.62 mm and 9.45 ± 0.65 mm, respectively) (1). In the present study, the HKL–SMM-Na combination reduced the MIC of SMM-Na by 8–32-fold, showed FICI values ≤0.5, produced bactericidal effects in time-kill assays, and was accompanied by increased membrane permeability, pronounced morphological damage, ATP depletion, and inhibition of biofilm formation. Thus, the HKL + SMM-Na synergy is consistent with an established paradigm in which membrane-active adjuvant-like compounds enhance antibiotic efficacy by compromising the bacterial envelope and core physiological functions. While some adjuvants, such as magnolol, act by inhibiting specific resistance enzymes like NDM-1 (43), and others like arbutin target virulence factors (44), our current data do not support a defined molecular target for HKL in this context. Whether HKL interacts with specific membrane components or proteins remains a valuable question for future, more mechanistic studies (45).

### 4.2 Effective *in vivo* protection is associated with pathogen control and host recovery

The protective effect of the low-dose SMM-Na + HKL combination was further evaluated in a *P. vannamei* challenge model. The selected low-dose combination treatment achieved the highest survival rate (86.6%), accompanied by a marked reduction in hepatopancreatic bacterial load. These *in vivo* results indicate that the SMM-Na + HKL regimen improved pathogen control under the experimental challenge conditions. The *in vitro* assays suggest that membrane-associated bacterial damage and energy depletion may contribute to the enhanced antibacterial activity of the combination. However, the *in vivo* outcome of dietary treatment is influenced by multiple factors, including feed intake, compound absorption, tissue exposure, pathogen burden, and host recovery status. Therefore, the *in vitro* and *in vivo* findings should be viewed as mutually supportive but not directly dose-equivalent. Further pharmacokinetic and pharmacodynamic studies will be useful to define tissue exposure levels of SMM-Na and HKL and to optimize the dosing strategy for practical aquaculture application. In addition to the reduced bacterial burden, improved survival was accompanied by changes in host homeostasis-related parameters. The infection-induced decline in THC, which may reflect hemocyte consumption, tissue recruitment, or infection-associated immune disturbance, was both delayed and less severe in the combination group, followed by a rapid recovery to levels near the negative control. Concurrently, the combination treatment was associated with increased transcription of multiple immune-related genes (*Alf*, *Tlr*, *Lec*, *Crustin*, *Lzm*, *CatB*) in the hepatopancreas and mucosal barrier genes (*Muc-1*, *Muc-4*, *Muc-5AC*, *Muc-19*) in the intestine. Together with the recovery of THC and reduced bacterial burden, these transcriptional changes provide additional evidence of immune- and barrier-associated host response recovery during treatment. Nevertheless, these qPCR-based data primarily reflect changes at the transcript level, and future studies incorporating protein-level validation or immune functional assays would help further define the biological significance of these responses. Related shrimp studies using phytogenic compound–antibiotic combinations, including San-Huang-San with enrofloxacin, florfenicol with chlorogenic acid, and Astragalus polysaccharide with florfenicol, have also reported improved survival and host immune-related parameters under bacterial challenge conditions (29, 46, 47). Accordingly, the attenuated histopathological damage observed in the hepatopancreas and intestine of the combination-treated shrimp is consistent with reduced pathogen burden and accompanying recovery of host tissue integrity. Future studies using expanded dose-matrix designs will be useful for quantitatively defining the contribution of each component and further optimizing the therapeutic window of the HKL + SMM-Na combination.

### 4.3 Treatment-associated shifts in intestinal microbiota during infection recovery

The SMM-Na + HKL combination was associated with detectable changes in the intestinal microbiota, which is closely linked to shrimp health. *V. parahaemolyticus* infection caused a dysbiotic state characterized by reduced microbial alpha diversity. The combination therapy increased alpha diversity indices relative to the infected control and was associated with a distinct microbial community profile. Notably, it suppressed the relative abundance of potentially pathogenic genera like *Vibrio* and *Motilimonas*. These changes are consistent with a treatment-associated shift of the gut-associated microbiota, characterized by increased microbial diversity, reduced dominance of potentially pathogenic genera, and altered predicted functional profiles during recovery from infection, rather than a simple nonspecific bactericidal effect. Similar microbiota-modulating effects towards improved health have been observed with other phytogenic supplements in shrimp (48, 49). Functionally, PICRUSt2 prediction indicated that the combination treatment was associated with group-dependent differences in predicted pathways, including those related to amino acid metabolism and xenobiotic biodegradation, as well as pathways linked to infectious diseases. These predicted functional profiles should be viewed as microbiota-associated functional potential inferred from 16S rRNA data. In the context of the reduced hepatopancreatic bacterial burden and improved tissue condition observed in the combination-treated shrimp, the intestinal microbiota changes likely represent part of the broader ecological and physiological recovery process following treatment. However, the present 16S rRNA-based analysis does not define whether these microbiota changes actively contributed to protection or occurred secondary to reduced pathogen pressure and host recovery. Further studies using microbiota-transfer, depletion, or reconstitution approaches would help clarify the functional contribution of the gut microbiota to treatment efficacy. This indicates that the therapeutic benefit may operate at the system level, encompassing pathogen clearance, host immunocompetence recovery, and microbiota-associated ecological changes. This multi-targeted response pattern is relevant to sustainable disease management, which requires strategies that address both the pathogen and the host’s overall physiological and ecological state (32, 47).

## 5 Conclusions

This study demonstrates that HKL can enhance the activity of SMM-Na against AHPND-causing *V. parahaemolyticus* under the tested conditions. The *in vitro* synergy was associated with HKL-related membrane damage and disruption of bacterial energy homeostasis, which is consistent with the action pattern of membrane-active antibiotic adjuvant-like compounds. In the *P. vannamei* challenge model, the low-dose SMM-Na + HKL combination improved survival and reduced hepatopancreatic bacterial burden, accompanied by attenuated tissue damage, recovery of hemocyte counts, increased transcription of immune and mucosal barrier-related genes, and treatment-associated changes in intestinal microbiota profiles. These findings support HKL as a promising natural-origin adjuvant candidate for enhancing SMM-Na efficacy against AHPND-causing *V. parahaemolyticus* in shrimp aquaculture. By improving the protective efficacy of low-dose SMM-Na under experimental challenge conditions, the HKL + SMM-Na strategy provides proof-of-concept evidence for a dose-sparing approach to AHPND control, while potentially reducing therapeutic SMM-Na input and associated environmental antimicrobial exposure. Future research should focus on elucidating potential molecular interactions of HKL, evaluating the efficacy and safety of this combination across different farming conditions and *Vibrio* strains, defining the pharmacokinetic/pharmacodynamic profiles of SMM-Na and HKL in shrimp, and optimizing delivery formulations to facilitate practical application.

## Data Availability

The bacterial genome sequence data generated in this study are available in GenBank under accession numbers CP187480–CP187485. The 16S rRNA amplicon sequencing raw data generated in this study have been deposited in the NCBI Sequence Read Archive under BioProject accession number PRJNA1479544 and SRA Study accession number SRP710549. The corresponding SRA Run accession numbers are SRR39214640–SRR39214646 and SRR39214648-SRR39214652.
