# Supplementary material for: Honokiol potentiates low-dose sulfamonomethoxine sodium against antimicrobial-resistant AHPND-causing Vibrio parahaemolyticus through bacterial membrane disruption and host recovery
Source: Front Immunol. 2026 Jun 30;17:1882728. doi: 10.3389/fimmu.2026.1882728 (PMC13364609; doi:10.3389/fimmu.2026.1882728)
Supplement: Supplementary file 1 [file DataSheet1.docx]

**
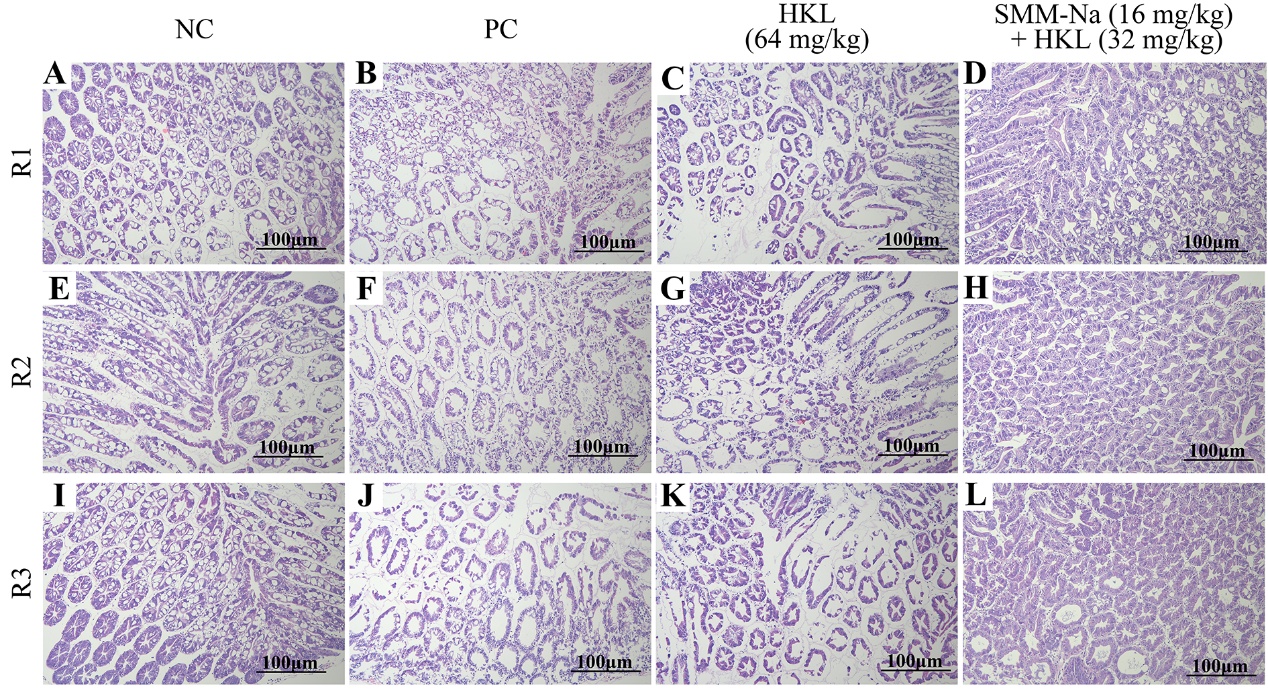
**

Figure S1. Representative H&E images of hepatopancreas used for histopathological scoring.

H&E-stained hepatopancreas sections/images used for histopathological scoring are shown. Groups include NC, PC, HKL (64 mg/kg), and SMM-Na (16 mg/kg) + HKL (32 mg/kg). R1–R3 indicate three scored sections/images per group. Semi-quantitative histopathological scores are summarized in Table S1. Scale bars, 100 μm.


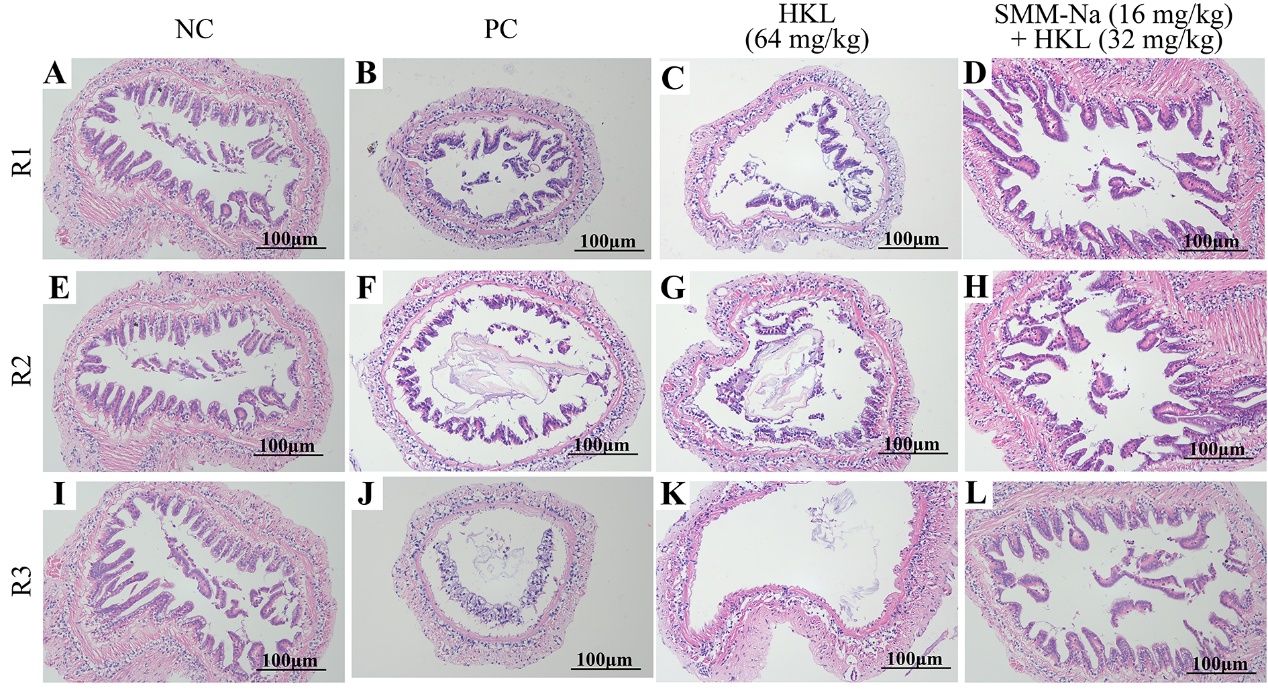


Figure S2. Representative H&E images of intestine used for histopathological scoring.

H&E-stained intestine sections/images used for histopathological scoring are shown. Groups include NC, PC, HKL (64 mg/kg), and SMM-Na (16 mg/kg) + HKL (32 mg/kg). R1–R3 indicate three scored sections/images per group. Semi-quantitative histopathological scores are summarized in Table S1. Scale bars, 100 μm.

Table S1 Semi-quantitative histopathological scores of hepatopancreas and intestine.

| Tissue | Group | R1 | R2 | R3 | Mean ± SD |
| --- | --- | --- | --- | --- | --- |
| Hepatopancreas | NC | 0 | 0 | 0 | 0.00 ± 0.00 |
| Hepatopancreas | PC | 3 | 3 | 4 | 3.33 ± 0.58 |
| Hepatopancreas | HKL | 3 | 2 | 3 | 2.67 ± 0.58 |
| Hepatopancreas | SMM-Na + HKL | 0 | 0 | 1 | 0.33 ± 0.58 |
| Intestine | NC | 0 | 0 | 0 | 0.00 ± 0.00 |
| Intestine | PC | 4 | 4 | 4 | 4.00 ± 0.00 |
| Intestine | HKL | 3 | 2 | 4 | 3.00 ± 1.00 |
| Intestine | SMM-Na + HKL | 1 | 1 | 1 | 1.00 ± 0.00 |

Note: R1–R3 represent three scored H&E sections/images per group. Histopathological lesions were scored on a 0–4 scale, with 0 indicating no obvious lesion and 4 indicating severe lesions. Data are presented as mean ± SD. NC, negative control; PC, positive control; HKL, honokiol; SMM-Na, sulfamonomethoxine sodium.
